# A gene signature linked to fibroblast differentiation for prognostic prediction of mesothelioma

**DOI:** 10.1186/s13578-023-01180-7

**Published:** 2024-03-10

**Authors:** Jun Liu, Yuwei Lu, Yifan Liu, Wei Zhang, Shuyuan Xian, Siqiao Wang, Zixuan Zheng, Ruoyi Lin, Minghao Jin, Mengyi Zhang, Weijin Qian, Jieling Tang, Bingnan Lu, Yiting Yang, Zichang Liu, Mingyu Qu, Haonan Ma, Xinru Wu, Zhengyan Chang, Jie Zhang, Yuan Zhang

**Affiliations:** 1 Department of Anesthesiology, Shanghai Pulmonary Hospital Affiliated to Tongji University, 507 Zheng Min Road, Shanghai, 200433 China; 2 Shanghai Jiao Tong University School of Medicine, Shanghai, 200025 China; 3 Department of Urology, Xinhua Hospital, Shanghai Jiaotong University School of Medicine, No. 1665 Kongjiang Road, Shanghai, 200092 China; 4 Department of Burn Surgery, The First Affiliated Hospital of Naval Medical University, Shanghai, China; 5 Research Unit of Key Techniques for Treatment of Burns and Combined Burns and Trauma Injury, Chinese Academy of Medical Sciences, Shanghai, China; 6 Tongji University School of Medicine, Shanghai, 200092 China; 7 Department of Pathology, School of Medicine, Shanghai Tenth People’s Hospital, Tongji University, 301 Yanchang Road, Shanghai, 200072 China; 8 Department of Gynecology, Shanghai First Maternity and Infant Hospital, Tongji University School of Medicine, 2699 Gaoke West Road, Shanghai, 201204 China; 9 Department of Pulmonary and Critical Care Medicine, Shanghai Pulmonary Hospital, Tongji University School of Medicine, Shanghai, 200433 China

**Keywords:** Mesothelioma, MESO, Single-cell RNA sequencing, Fibroblast, Differentiation

## Abstract

**Background:**

Malignant mesothelioma is a type of infrequent tumor that is substantially related to asbestos exposure and has a terrible prognosis. We tried to produce a fibroblast differentiation-related gene set for creating a novel classification and prognostic prediction model of MESO.

**Method:**

Three databases, including NCBI-GEO, TCGA, and MET-500, separately provide single-cell RNA sequencing data, bulk RNA sequencing profiles of MESO, and RNA sequencing information on bone metastatic tumors. Dimensionality reduction and clustering analysis were leveraged to acquire fibroblast subtypes in the MESO microenvironment. The fibroblast differentiation-related genes (FDGs), which were associated with survival and subsequently utilized to generate the MESO categorization and prognostic prediction model, were selected in combination with pseudotime analysis and survival information from the TCGA database. Then, regulatory network was constructed for each MESO subtype, and candidate inhibitors were predicted. Clinical specimens were collected for further validation.

**Result:**

A total of six fibroblast subtypes, three differentiation states, and 39 FDGs were identified. Based on the expression level of FDGs, three MESO subtypes were distinguished in the fibroblast differentiation-based classification (FDBC). In the multivariate prognostic prediction model, the risk score that was dependent on the expression level of several important FDGs, was verified to be an independently effective prognostic factor and worked well in internal cohorts. Finally, we predicted 24 potential drugs for the treatment of MESO. Moreover, immunohistochemical staining and statistical analysis provided further validation.

**Conclusion:**

Fibroblast differentiation-related genes (FDGs), especially those in low-differentiation states, might participate in the proliferation and invasion of MESO. Hopefully, the raised clinical subtyping of MESO would provide references for clinical practitioners.

**Supplementary Information:**

The online version contains supplementary material available at 10.1186/s13578-023-01180-7.

## Introduction

Malignant mesothelioma is an uncommon but aggressive and lethal malignancy that arises from mesothelial cells of plasma membrane tissues, such as the pleura, peritoneum, and pericardium, with a median overall survival of approximately 8 months [1]. During 1999–2015, malignant mesothelioma contributed to 45,221 deaths in the United States [2]. Although asbestos has been outlawed in some countries to avoid asbestos exposure which is the prime etiology of MESO, the incidence and disease burden will still grow in the future due to the long latency (around 20 to 40 years) of MESO following exposure [3–5], drawing experts’ attention. What’s more, effective therapies for MESO are scarce, which can be partly attributed to its relative resistance to radiotherapy and chemotherapy and the fact that a considerable proportion of the tumors cannot be radically resected. The situation makes it difficult to reverse the poor prognosis of MESO. Moreover, the current stage-based categorization of MESO is insufficient for precise diagnosis and treatment [6], which raises the requirement for a new classification and risk assessment method. Collectively, it’s imperative to find more prognosis-related factors, which may improve the precise stratification of MESO diagnosis and offer some novel insights into the pursuit of therapeutic targets for MESO.

In recent decades, the interaction between malignant cells and stroma which consists of immune cells, fibroblasts, capillaries, extracellular matrix (ECM) and so on, has been considered to take part in tumor progression and invasion [7]. While normal fibroblasts were found to inhibit the transformation of malignant cell phenotypes [8], cancer-associated fibroblasts (CAFs) exhibited the protumorigenic effect [9], implicating the heterogeneity of fibroblasts in tumors. CAF, a type of activated fibroblast with high heterogeneity, functions as an important protumor player via multiple mechanisms, such as mediating angiogenesis with increasing CXCL-12 secretion [10], activating heat shock factor 1 (HSF1) [11] and impacting ECM stiffness to create tracks for malignant cell migration [12]. Moreover, it has been reported that tumor-associated fibroblasts can boost the progression of malignant pleura mesothelioma through a cytokine network involving fibroblast growth factor-2 (FGF-2), platelet-derived growth factor-AA (PDGF-AA), and hepatocyte growth factor (HGF) [13]. In summary, the development of cancer is significantly influenced by fibroblasts. Accordingly, we deem that it is valuable to search for the heterogeneity and differentiation of fibroblasts in MESO.

In the study, we leveraged the single-cell RNA sequencing profiles of MESO to identify several fibroblast subtypes and obtain their differentiation states. Utilizing bulk RNA sequencing and survival information of MESO samples from the TCGA database, we obtained 39 fibroblast differentiation-related genes (FDGs), which were associated with survival as well. Based on the FDGs, we tried to construct an original classification and a prognostic prediction model for MESO and expected them to have a decent prognostic performance. On this basis, we made inhibitor predictions as well as proposed some assumptions for new therapeutic targets. Last but not least, except for in silico analysis, clinical specimens were extracted for wet experiment validation.

## Methods

### Data source

This study obtained the approval of the Ethics Committee of the First Affiliated Hospital of Naval Medical University. The primary single-cell RNA sequencing profiles for MESO were downloaded from the Gene Expression Omnibus (GEO) database (http://www.ncbi.nlm.nih.gov/geo/) (GEO accession: GSE201925) [14]. The bulk RNA sequencing data and clinical information of MESO, such as demographic statistics of patients, TNM stages, and survival time, were collected through the TCGA database (https://tcga-data.nci.nih.gov/tcga/) for multi-omics analysis. We also retrieved the information on metastatic MESO from MET500 (https://www.pathology.med.umich.edu/mctp/mi-oncoseq-study) and compared it with data on primary MESO derived from the TCGA database.

### Bioinformation tools

The single-cell RNA sequencing data was preprocessed using the 10× Genomics Chromium System (https://www.10xgenomics.com/instruments/chromium-x-series) according to the manufacturer’s protocol. Subsequent analyses were conducted by R version 4.1.3 software (Institute for Statistics and Mathematics, Vienna, Austria; www.r-project.org) and various R packages which we mentioned later.

### Comprehensive bioinformatics analysis

#### Primary analysis of single-cell RNA sequencing data

Quality control, normalization, principal component analysis (PCA), t-Distributed Stochastic Neighbor Embedding (tSNE) analysis, differential expression analysis, and cell communication analysis were performed on the raw single-cell RNA sequencing data of three MESO samples obtained from GEO.

We turned the raw data into fastq files using the Cell Ranger software package (v3.0, http://10xgenomics.com/), which were then refined into the gene expression matrix using cellranger count software. The subsequent steps, including quality control, dimensional reduction, clustering, and differentially expressed analysis, were carried out using the Seurat R package (version 4.0.6; https://satijalab.org/seurat/) [15]. We normalized the data after removing empty droplets, droplets containing low-quality cells, and droplets containing two or more cells. The top 2000 highly variable genes were then chosen and underwent linear dimensionality reduction (PCA) to check dataset dimensionality and non-linear dimensionality reduction (tSNE) to visualize and study datasets. Meanwhile, we obtained cell clusters in tSNE space using the graph-based clustering technique. We identified the cell types of these clusters by identifying the marker genes of each cell cluster using differential expression analysis. An absolute log2 (fold change) > 0.50 and an adjusted p-value < 0.05 were used for differential expression analysis.

Following that, we retrieved the scRNA-seq profiles of fibroblasts based on the previously identified cell clusters and repeated the prior analytic stages to further filter out cell subtypes. Important ligand and receptor pairs that were differently expressed in cells were caught by the iTALK package in order to identify possible signaling routes between cell clusters [16]. Afterward, we used gene set variation analysis (GSVA) to identify enriched pathways within each cluster.

#### Identification of fibroblast differential genes (FDGs) and consensus clustering

The Monocle2 R package was operated to conduct the pseudotime analysis of fibroblasts, identifying the genes that were possibly associated with the differentiation fate of fibroblasts [17]. Then we filtered out the genes that were expressed differentially with statistical significance (P value < 0.001) among fibroblast subtypes, as monocle2_sig genes.

Furthermore, via univariate Cox regression and Kaplan–Meier survival analysis of bulk RNA-seq profiles and survival data from the TCGA database, we obtained uniCox_sig genes and KM_sig genes with statistical significance (p value < 0.001).

The genes that occurred in the monocle2_sig gene group, the uniCox_sig gene group, and the KM_sig gene group at the same time were assumed to be fibroblast differential genes (FDGs).

For a further multi-omics study, we divided samples into various clusters according to the differential expression of FDGs using the ConsensusClusterPlus R program [18]. To confirm the optimal number of clusters, we created heatmaps of the consensus matrix with different k values. We also utilized the Elbow approach to verify the result. After clustering, Kaplan–Meier survival analysis was used to analyze the prognosis differences of these clusters, and the association between the PCA scores and the prognosis circumstances was explored as well.

#### PCA score and the clusters: multi-omics association

The samples’ data we processed in the multi-omics analysis was searched from the TCGA database. Afterwards, the multi-omics association of the PCA score and the clusters was explored, involving genomics and transcriptomics. According to the PCA score, we separated samples into two sets: the low-PCA_score set and the high-PCA_score set.

With the ‘Maftools’ R package, we presented conditions of gene mutation in two sets of samples and showed the distribution of tumor mutation burden (TMB) and MSI [19]. The Pearson correlation test was utilized to evaluate the correlation between the PCA score and TMB/MSI. The statistical significance was defined as a P value < 0.05. What’s more, we calculated the TMB score and regrouped the samples into four groups, combining the PCA score and the TMB score. Implementing the Kaplan–Meier survival analysis, we tried to discover the differences in survival conditions in the four groups. The analysis of copy number variations (CNV) of FDGs was performed as well, and the result was visualized.

The transcriptomics analysis was mainly focused on the immune infiltration landscape. With Single-Sample Gene Set Enrichment Analysis (ssGSEA), we detected infiltration degrees of 23 immune cells in three clusters, which were gained from consensus clustering [20]. We also compared the expression levels between the low-PCA_score set and the high-PCA_score set. Lastly, the correlation between immune infiltration and the PCA score was assessed through co-expression analysis.

#### Differential expression analysis between primary and bone-metastatic tumors

We compared the expression conditions of FDGs and transcription factors (TFs) between the primary MESO data from TCGA and bone-metastatic tumors from MET 500. The analysis was carried out by the limma method, and the differential expression was regarded as an absolute log2 (fold change) > 1.0 and an adjusted P value < 0.05 [21]. GSVA was also applied to compare the differences in enriched pathways between primary MESO and MESO with bone metastases.

#### Building a prognostic prediction model

The Molecular Signatures Database (MSigDB) (https://github.com/tomastokar/gsoap) was then used to perform over-representation analysis (ORA) on gene sets enriched in FDGs. The hazard ratios of the FDGs were calculated by the univariate Cox regression. We used the glmnet R package to create a predictive model, filtering out six differentiation-related genes from the FDGs with the least absolute shrinkage and selection operator (Lasso) regression [22]. These genes were input into the multivariate Cox model. With the regression coefficients of these genes determined, we calculated the risk score of each sample and sorted the samples into two groups: the high-risk group and the low-risk group. Next, we contrasted the prognosis conditions of the two groups with survival data, utilizing Kaplan–Meier survival analysis. The expression conditions of these filtered genes in the two groups were compared as well. Finally, the model’s accuracy was measured using the receiver operator characteristic (ROC) curve.

The formula of risk score is as follows:

$${risk}\;{score_m}=\sum_{i=1}^{n} {\beta_i} \times {Gene_i}$$

where m: the number of samples; n: the number of the filtered genes; Gene_i_: the normalized expression level; β_i_: the corresponding regression coefficient of each filtered gene.

#### Immune infiltration analysis

With nu support vector regression, the CIBERSORT displayed the infiltration proportion of 22 immune cells in both the high-risk group and the low-risk group [23]. Meanwhile, the statistically significant difference in the infiltration proportions of immune cells between the two groups was validated through the T-test. Following that, with ssGSEA, we tried to find the deviations in immune functions between the two groups. Lastly, we combined the survival data with immune cells and functions to explore whether they had connections, applying Kaplan–Meier survival analysis.

#### Investigation of regulatory networks and ATAC analysis

To tentatively probe into the important regulatory mechanisms in the three clusters, we attempt to establish the regulatory networks involving important genes, transcription factors (TF), pathways, immune cells, and RPPA. To ensure statistical significance, we used Spearman correlation analysis and Pearson correlation analysis with R > 0.300 and a P value of 0.0001.

The chromatin accessibility at the sites of the genes in the regulatory networks was accessed by ATAC-seq analysis, exploring the transcriptional mechanism of these genes.

#### Identification of inhibitors

As the pRRophetic R package can predict the chemotherapeutic response of samples, we employed it to estimate the drug sensitivity of each cluster with the Cancer Cell Line Encyclopedia (CCLE) and output the results in the form of violin plots [24]. The drugs that revealed a statistically significant difference in sensitivity between the cluster with the best survival conditions and the one with the worst.

### Clinical specimen validation

#### Data source

The study was approved by the Ethics Committee of the First Affiliated Hospital of Naval Medical University. Due to the rarity of MESO, only a total of 11 tumor samples derived from 11 patients diagnosed with malignant MESO from January 1, 2020 to December 31, 2022 in the First Affiliated Hospital of Naval Medical University were collected for clinical validation. Patients’ characteristics, including age, gender, T, N, M, stage, PS scores, and pathological reports, were as well retrieved.

#### Immunohistochemical staining validation

The 11 tumor tissues were initially fixed, embedded and sliced into paraffin samples. Then the samples were dewaxed and rehydrated, after which antigen retrieval was performed to reveal the antigens (markers of each MESO subtype, including SERPINE, UBE2C, CDC20, HP, and CFB) via EDTA (pH = 9.0) or citric acid (pH = 6.0) antigen-retrieving buffers. Afterwards, to prevent non-specific binding of antibodies, the samples were incubated in a 3% hydrogen peroxide solution and subsequently washed in a PBS solution. Following that, endogenous peroxidase was blocked by treating the samples with 3% BSA or rabbit serum. Then the primary antibodies were added to bind to the antigens, subsequently, the secondary antibodies, marked by the enzyme HRP, were added, which would bind to the primary antibodies if the primary antibodies were bound. Then the specific substrate DAB for HRP was then added for color development. If the antigens of interest were present, the DAB would react with HRP, and this reaction would provide a colour change which could be visualized. In order to aid visualization, a counterstain by adding haematoxylin was utilized to provide contrast and highlight of the nuclei. Then the samples were dehydrated using a series of graded ethanol and all the slides were imaged via a light microscope. Last but not least, several professional pathologists were invited to read and score these slides.

#### Retrospective study validation

Statistical analyses of the pathological and clinical information of the 11 MESO patients were performed for further validation. The differentiation degree and immune infiltration of the samples were evaluated by several professional pathologists, based on which MESO patients were divided into well- and poorly-differentiated subgroups, or low- and high-immune infiltration subgroups. Moreover, the metastasis data of MESO patients was extracted to categorize them into metastatic and non-metastatic subgroups. Subsequently, Pearson Chi-square tests were conducted on different MESO subtypes with good or poor differentiation, low- or high-immune infiltration, and metastasis or not.

## Results

### Fibroblasts identified in the tumor microenvironment

**Fig. 1:**
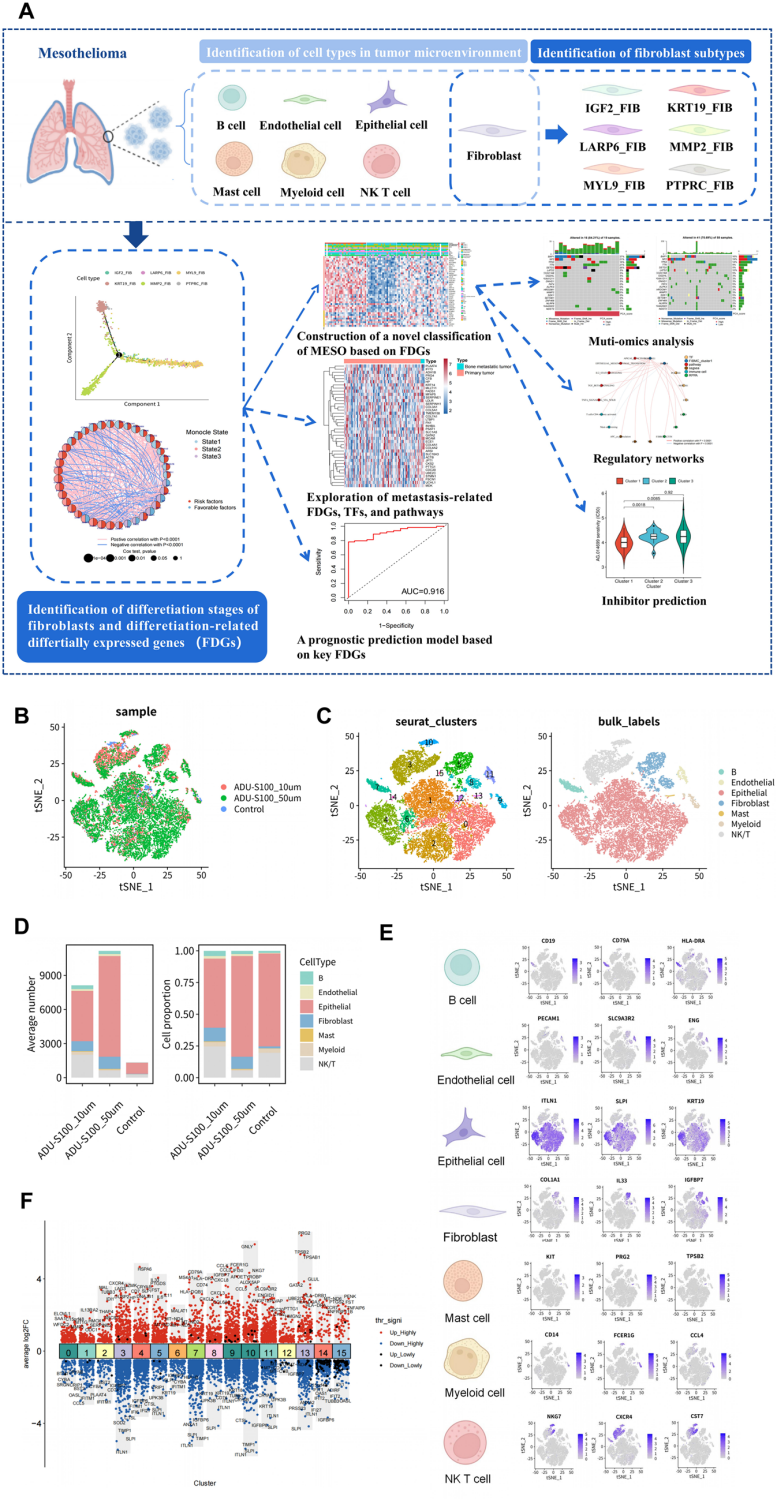
The gene expression landscape of cells in MESO microenvironment.** A** The workflow of bioinformation analysis in the study. **B** The tSNE plot of single cell sequencing data from three samples (ADU-S100_10um, ADU-S100_50um, and control). **C** Identification of 15 cell clusters and seven cell types (B cell, endothelial cell, epithelial cell, fibroblast, mast cell, myeloid cell, and NK/T cell) in the tSNE analysis with cell markers. **D** The histogram of average number and proportion of each cell type in the three samples. **E** Respective cell markers of each cell type. **F** Gene differential expression pattern of the 15 cell clusters

We acquired gene expression profiles of three MESO samples (ADU-S100_10um, ADU-S100_50um, and control) at the single cell level from the Gene Expression Omnibus (GEO) database. A total of 14,347 single-nucleus RNA sequencing data were obtained after stringent filtration and control for subsequent analysis. The detailed procedure of the study was visualized in Fig. 1A and Additional file 1: Fig. S1. To identify the key stromal cells that may have regulatory functions in tumor progression, we utilized dimension reduction analysis and discovered 15 seurat clusters, which were categorized into seven cell types (B cell, endothelial cell, epithelial cell, fibroblast, mast cell, myeloid cell, and NK/T cell) in accordance with cell markers, in the tumor microenvironment (Fig. 1B, C). The expression conditions of cell type marker genes, which assisted in the classification of cell types, were shown in Fig. 1E. What’s more, the average number and cell proportion of the seven cell types in the three samples were depicted in Fig. 1D, illustrating the highest number of the epithelial cell, followed by the NK/T cell. In the differential expression analysis of the 15 seurat clusters, we found that some clusters that were recognized as the same cell types showed a similar differential expression landscape, exhibiting the rationality of the cell type identification (Fig. 1F). We further explored the differential expression conditions of the seven cell types, visualizing the expression of four classic cell markers (CD3D, LYZ, CD79A, and VWF) and listing the top five differentially expressed genes in each cell type, respectively, in Additional file 1: Fig. S2A, B. As CD3D, LYZ, CD79A, and VWF were highly expressed in NK/T cells, myeloid cells, endothelial cells and B cells, respectively, their expression was low in the other cells, illustrating the accuracy of the cell type identification. Subsequently, to figure out the relationship and interactions between fibroblasts and other cells, a cell communication network and ligand-receptor interactions among seven cell types were established. As shown in Additional file 1: Fig. S2C, D, fibroblast lay at the heart of the communication network and possessed the most frequent connections with the other six cell types, emphasizing its momentous role in regulating the biological behavior of MESO. Moreover, cell cycle score and cell cycle distribution based on the tSNE showed fibroblasts were uniformly distributed across the G1, S, and G2M phases of the cell cycle (Additional file 1: Fig. S2E, F), partly reflecting the heterogeneity among fibroblasts.

**Fig. 2:**
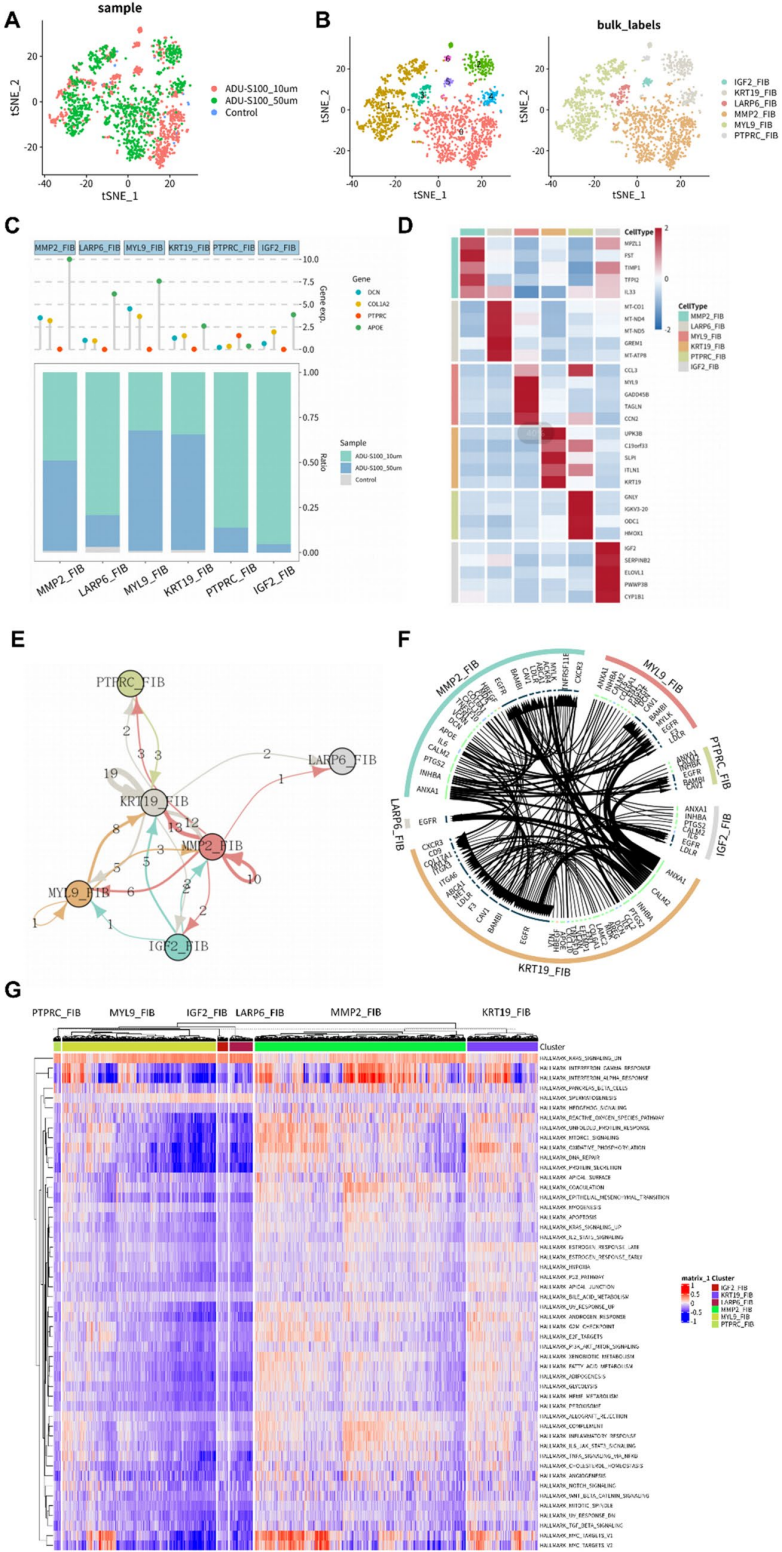
Identification and features’ capture of fibroblast subtypes in MESO environment.** A** The tSNE plot of fibroblasts which were identified in the MESO environment in the last step. **B** Recognition of seven fibroblast clusters and six fibroblast subtypes (IGF_FIB, KRT19_FIB, LARP6_FIB, MMP2_FIB, MYL9_FIB and PTPRC_FIB) in the tSNE analysis. **C** The expression levels of four classic cell markers (DCN, COL1A2, PTPRC and APOE) and proportion of the three samples in each fibroblast subtype. **D** The top four or five differentially expressed genes in each fibroblast subtype. **E** The cell communication network of fibroblast subtypes. **F** The prediction of the interactions between each fibroblast subtype based on the ligand-receptor interactions. **G** The heatmap of pathway enrichment conditions of the six fibroblast subtypes based on GSEA analysis

Among the three MESO samples, 1969 fibroblasts were identified (Fig. 2A), from which we derived six fibroblast subtypes through dimension reduction analysis and clustering (Fig. 2B). Figure 2C displayed the expression level of four cell markers in each subtype. In addition to the archetypal fibroblast markers, DCN and COL1A2, the expression of APOE was generally high. We also listed the top four or five differentially expressed genes in each subtype in the form of a heatmap (Fig. 2D). The cell communication network and ligand-receptor interactions among six subtypes showed the central position of KRT19_FIB and MMP2_FIB among all the subtypes and the tight link between EFGR and both CALM2 and ANXA1 (Fig. 2E, F). In order to further explore the functions of each subtype, we performed GSVA based on variation scores of gene sets in each fibroblast, utilizing the Hallmark pathway database, revealing that all the subtypes were prevalently enriched in hallmark KRAS signaling dn, and both KRT19_FIB and MMP2_FIB had relatively high enrichment of immune-related hallmark gene sets (hallmark interferon gamma response and hallmark interferon alpha response) and proliferation-related hallmark gene sets (hallmark MYC targets V1 and hallmark MYC targets V2) [25], which implied that these two subtypes might have higher proliferative potential, compared to other subtypes (Fig. 2G). Therefore, among all six subtypes, KRT19_FIB and MMP2_FIB deserved more attention.

### The differentiation states of fibroblast subtypes and differentiation-related fibroblast differential genes (FDGs)

**Fig. 3:**
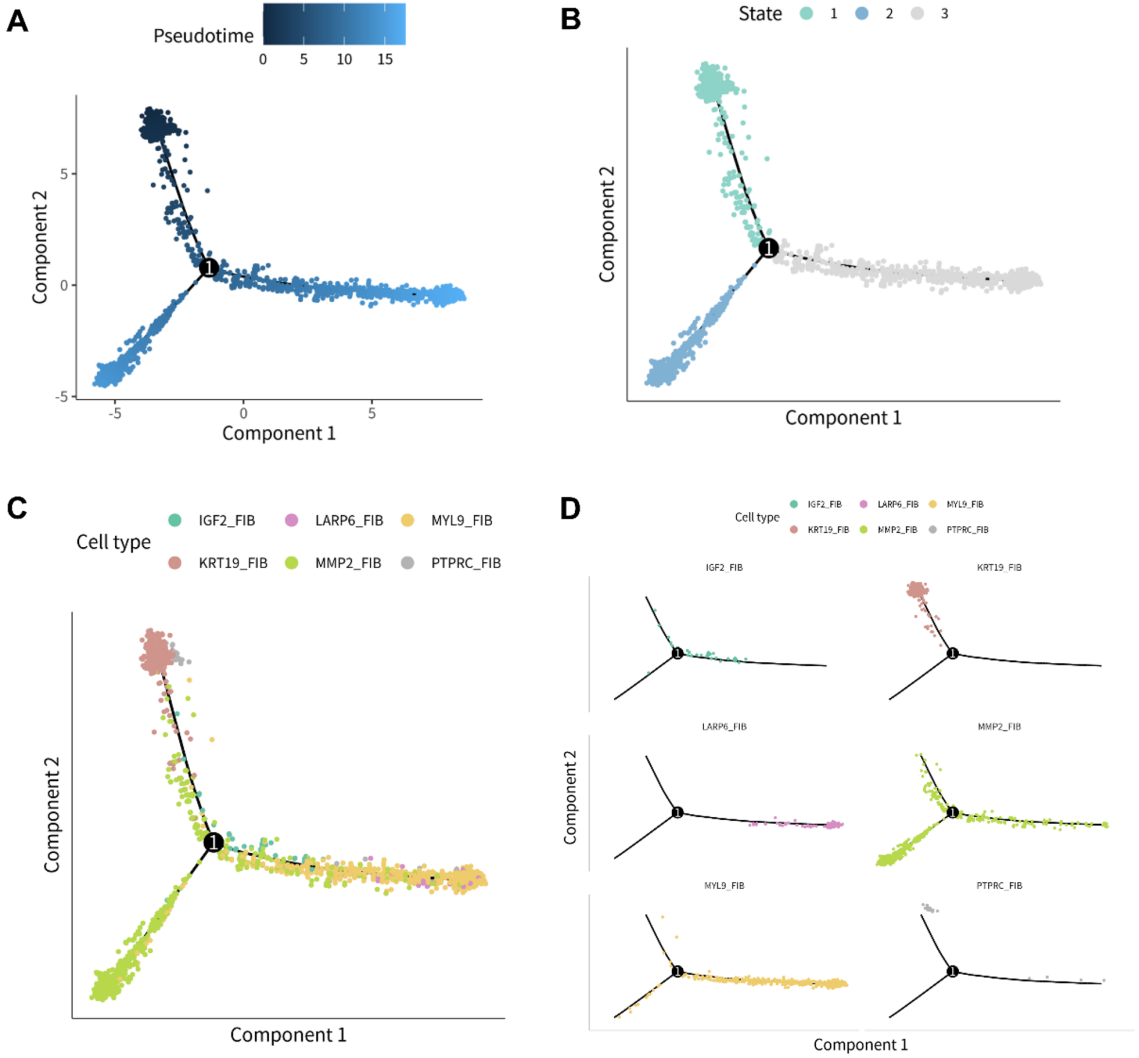
The pseudotime analysis of fibroblasts. **A** The differentiation trajectory of fibroblasts, in which the variation in shade of color standed for the passage of time. **B** The three differentiation states (state 1, state 2 and state 3) distinguished from the differentiation trajectory. **C** The overview map of the six fibroblast subtypes’ distribution in the differentiation trajectory. **D **The individual distribution condition of the six fibroblast subtypes in the differentiation trajectory, labeled with different colors

Monocle 2 and tSNE were utilized in the process. With 4 tSNE cell clusters recognized (Additional file 1: Fig. S3A), the differentiation trajectory of the fibroblast was visualized. In Fig. 3A, which illustrated the chronological order of the trajectory, the gradation of color represented the time cells took to differentiate. A total of three differentiation states were captured, of which state 1 was the earliest stage and was then separated into states 2 and 3 (Fig. 3B). The distribution of the six subtypes in the trajectory was presented in Fig. 3C, D. While IGF2_FIB, LARP6_FIB, and MYL9_FIB were mainly located in state 3, which implied relatively mature differentiation, KRT19_FIB and PTPRC_FIB were mostly distributed in state 1, a hypodifferentiated stage. The distribution of MMP2_FIB was dispersive, suggesting multiple differentiation states. Consistent with the distribution of KRT19_FIB in state 1, the expression of the top five differentially expressed genes of KRT19_FIB, including C19orf33, ITLN1, KRT19, SLPI, and UPK3B, was extremely concentrated in state 1, especially at the origin of the trajectory (Additional file 1: Fig. S3B). What’s more, the expression of these five genes in the origin was primarily derived from KRT19_FIB (Additional file 1: Fig. S3C), and their expression conditions in the three MESO samples were exhibited in Additional file 1: Fig. S3D. Therefore, the differentiation degree of fibroblasts in KRT19_FIB might be low.

**Fig. 4:**
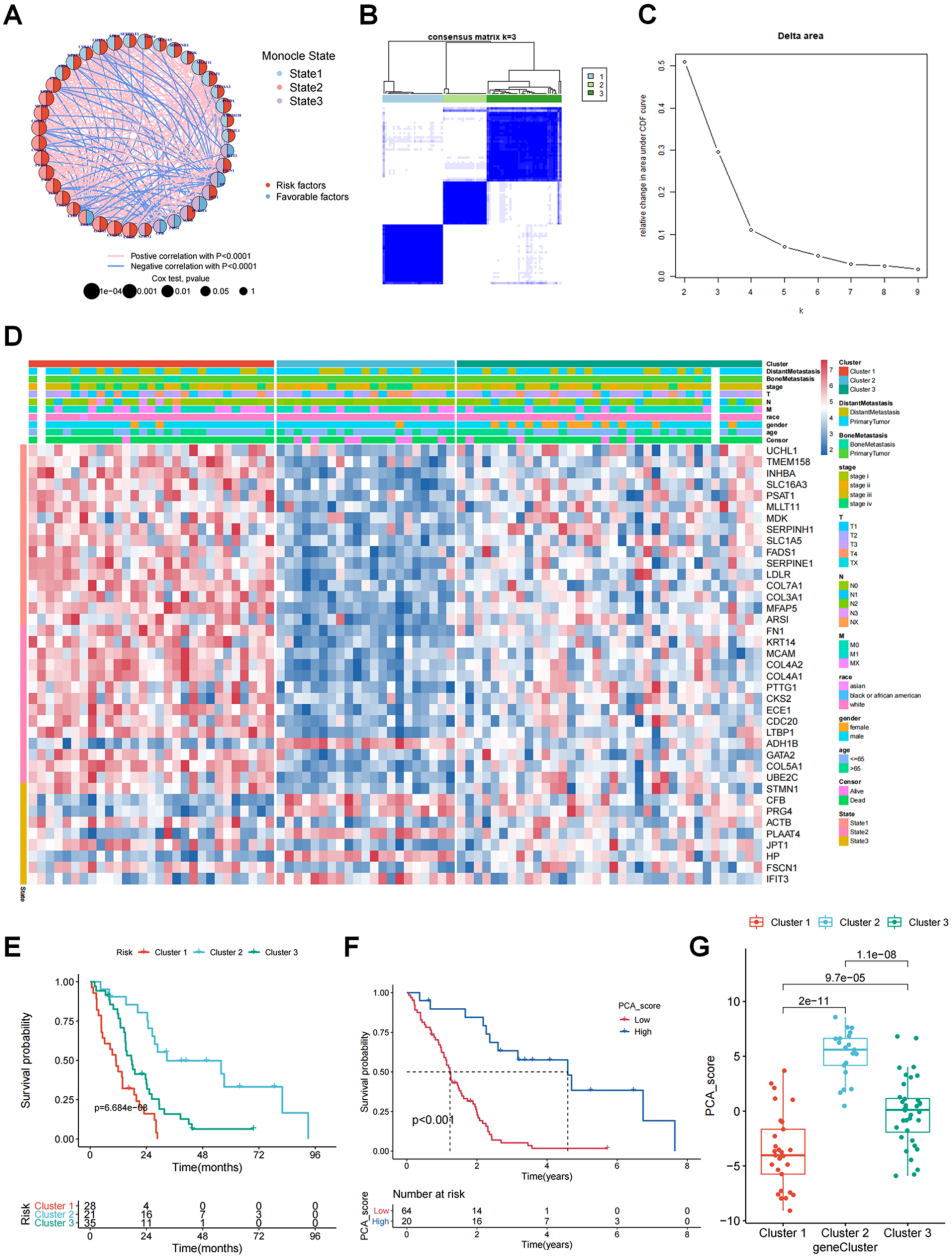
Construction of fibroblast differentiation-based classification of MESO patients in TCGA cohort.** A** The correlation network of 39 FDGs. The colors of the left half of 39 circles which correspond to the 39 FDGs, figured out the differentiation states of the genes, while the colors of the other side described the genetic effects on survival (Red: high expression was harmful for survival; Blue: high expression was favorable for survival). The size of circles was inversely proportional to the p value of Cox regression analysis. The links between circles displayed the relationship between genes where red referred to the positive correlation and blue indicated the negative correlation with remarkable statistical significance (p < 0.0001). **B** The Consensus matrix of k = 3. **C** The CDF curve and the delta area plot. **D** The cluster heatmap of FDGs expression in the patients’ MESO samples, based on bulk RNA-seq data from TCGA database, identifying three clusters. The clinical information of the patients in the cohort, including survival outcome, age, gender, race, TNM stage and metastatic condition were listed as well. The color in the heatmap from blue to red demonstrated the progression from low expression to high expression. **E** The comparison of survival conditions of the three clusters with the Kaplan–Meier curve. **F** The Kaplan–Meier survival analysis of the MESO patients in the high PCA score group and the low PCA score group. **G** The boxplot which displayed the distribution of the PCA scores of patients in the three clusters

Subsequently, we aimed to catch the differentiation-related fibroblast differential genes (FDGs) among the six subtypes. With the Monocle2 single-cell analysis toolset, 644 genes that were differentially expressed in the three differentiation states (p < 0.001) were extracted and shaped into the monocle2_sig gene set. On the basis of the bulk RNA-seq data and the clinical information of MESO patients recorded in the TCGA database, survival analysis was carried out using the Kaplan–Meier technique and the univariate Cox regression model to detect the survival-related genes and eventually form the KM_sig gene set (81 genes with p < 0.001) and the uniCox_sig gene set (150 genes with p < 0.001). Overlapping the three gene sets, a total of 39 genes in the intersection of the three gene sets were screened out and defined as fibroblast differentiation-related genes (FDGs) (Additional file 1: Table S1). According to the survival curves of the 39 FDGs (Additional file 1: Fig. S4), high expression of ADH1B, CFB, PRG4, PLAAT4, HP, and IFIT3 was favorable to survival, while the other 33 genes were considered risk factors. What’s more, five of the six favorable factors (CFB, PRG4, PLAAT4, HP, and IFIT3) were represented in state 3, as all 16 FDGs correlated with state 1 were marked as risk factors (Fig. 4A). Consequently, we deduced that in the MESO tumor microenvironment, fibroblasts with high degrees of differentiation might help to improve prognosis, whereas fibroblasts with low degrees of differentiation might have the opposite effect. Based on this inference, KRT19_FIB might be associated with an adverse prognosis.

### Construction of a fibroblast differentiation-based classification (FDBC) of MESO

Depending on pseudotime analysis to identify the dynamics of gene expression over the course of consecutive cell development, we tried to seek out the different differentiation states of fibroblasts and differentiation characteristics of each subtype.

In order to explore the clinical value of FDGs, we obtained the bulk RNA-seq data and clinical information of MESO patients from the TCGA database and divided these patients into different classifications on the basis of their expression of FDGs, utilizing consensus clustering. Combining the heatmaps of the consensus clustering matrix, the CDF curve, and the delta area plot, three was considered the optimal k value, producing three clusters (Fig. 4B, C). As shown in Fig. 4D, cluster 1 highly expressed the genes in states 1 and 2, while cluster 2 had relatively high expression of state 3 genes (covering CFB, PRG4, PLAAT4, HP, and IFIT3), which suggested that cluster 1 might be associated with lowly differentiated fibroblasts and the fibroblasts in cluster 2 trended to be highly differentiated. The differential expression of all the FDGs was statistically significant (Additional file 1: Fig. S5C). Therefore, we designated cluster 1 “Lowly Differentiated Fibroblast-Related Mesothelioma (LDFM),” cluster 2 “Highly Differentiated Fibroblast-Related Mesothelioma (HDFM),” and cluster 3 “Moderately Differentiated Fibroblast-Related Mesothelioma (MDFM)”. Among the three clusters, the survival result of HDFM was remarkably better than that of LDFM and MDFM, while that of LDFM was the worst, embodying the prognostic value of the novel classification (Fig. 4E). We further calculated the PCA score of each patient based on 39 FDGs and divided patients into two PCA groups (low-score group and high-score group) (Additional file 1: Fig. S5A, B). Since the survival probability of patients in the high-score group was commonly higher than that of those in the low-score group, the PCA score probably had a positive correlation with survival (Fig. 4F). Meanwhile, we found that the PCA scores of patients in HDFM were generally higher than those in LDFM and MDFM (Fig. 4G). All the patients in LDFM flowed to the low-score group, and HDFM comprised a large proportion of the high-score group (Additional file 1: Fig. S5B). Accordingly, the novel classification might be an effective indicator for prognostic prediction to improve patient management.

### The association between multi-omics alterations and FDGs

We performed multi-omics analysis in conjunction with the PCA score and the novel classification to better explain the prognostic difference caused by the differential expression of FDGs. This included the gene mutation landscape, tumor mutation burden (TMB), copy number variations (CNV), microsatellite instability (MSI), and immune infiltration. Although the major mutation genes were similar between the two PCA score groups, more gene types were discovered to mutate in the low-score group, such as FAT4, ALPK3, SDK1, SETDB1, and so on (Additional file 1: Fig. S6A), revealing the more aberrant biological activities of tumors in the low-score group to some extent. After that, we attempted to find out whether TMB and MSI changed along with the increase in PCA score. Unfortunately, the negative correlations between PCA score and both TMB (R = − 0.15, p = 0.18), and MSI (R = − 0.19, p = 0.084) were without statistical significance (Additional file 1: Fig. S6B, F). Uniting PCA score and TMB, the survival conditions of the low-TMB + high-PCA score group, the high-TMB + high-PCA score group, the low-TMB + low-PCA score group, and the high-TMB + low-PCA score group became worse in order (Additional file 1: Fig. S6C), which demonstrated the priority of PCA score over TMB in the survival assessment of MESO. Among the CNV alterations of 39 FDGs, the loss frequency of TMEM158 and COL7A1, which were respectively located on chromosomes 3 and 13, was arrestingly high (Additional file 1: Fig. S6D, F). Through comparing the immune infiltration of LDFM, HDFM, and MDFM, we noticed that the activated CD4+ T cells, CD56-dim natural killer cells, regulatory T cells, and type 2 T helper cells might have a tendency to gather in LDFM (Additional file 1: Fig. S7A), which is in line with the negative association between these cells and PCA score (Additional file 1: Fig. S7C). The expression of PD-L1 was not significantly different between the two groups (Additional file 1: Fig. S7B). On account of the vital role of immune cells in the anti-tumor and pro-tumor processes, the difference in the immune infiltration patterns of LDFM, HDFM, and MDFM may have contributed to the prognostic difference between them.

### Investigation of the genes, transcription factors (TFs), and signaling pathways linked to Metastasis

Differential expression analysis was performed, contrasting RNA-seq data of primary tumors from the TCGA database with data of bone metastatic tumors from the MET500 database. The heatmap and volcano plot in Additional file 1: Fig. S8A visualized the expression patterns of every FDG in primary and metastatic tumors. There were 8 overexpressed FDGs, including PSAT1, FADS1, MFAP5, LDLR, COL4A1, COL4A2, MCAM, and KRT14 (four in state 1 and four in state 2), and 2 downregulated FDGs, including HP and CFB (all in state 3), in metastatic tumors (Additional file 1: Fig. S8B). Most of the upregulation and downregulation of transcription factors occurred without statistical significance (Additional file 1: Fig. S8C). Ultimately, we compared the enrichment degree of 50 typical pathways in primary and metastatic tumors and observed 49 pathways that were upregulated in metastasis, including the apical junction, epithelial-to-mesenchymal transition (EMT), IL2-STAT5 signaling, G2M checkpoint, E2F targets, TGF beta signaling, Hedgehog signaling, and so on (Additional file 1: Fig. S8D). These genes and pathways might play a role in the advancement of MESO as motivators or suppressors.

### Establishment and internal verification of a novel prognostic prediction model

**Fig. 5:**
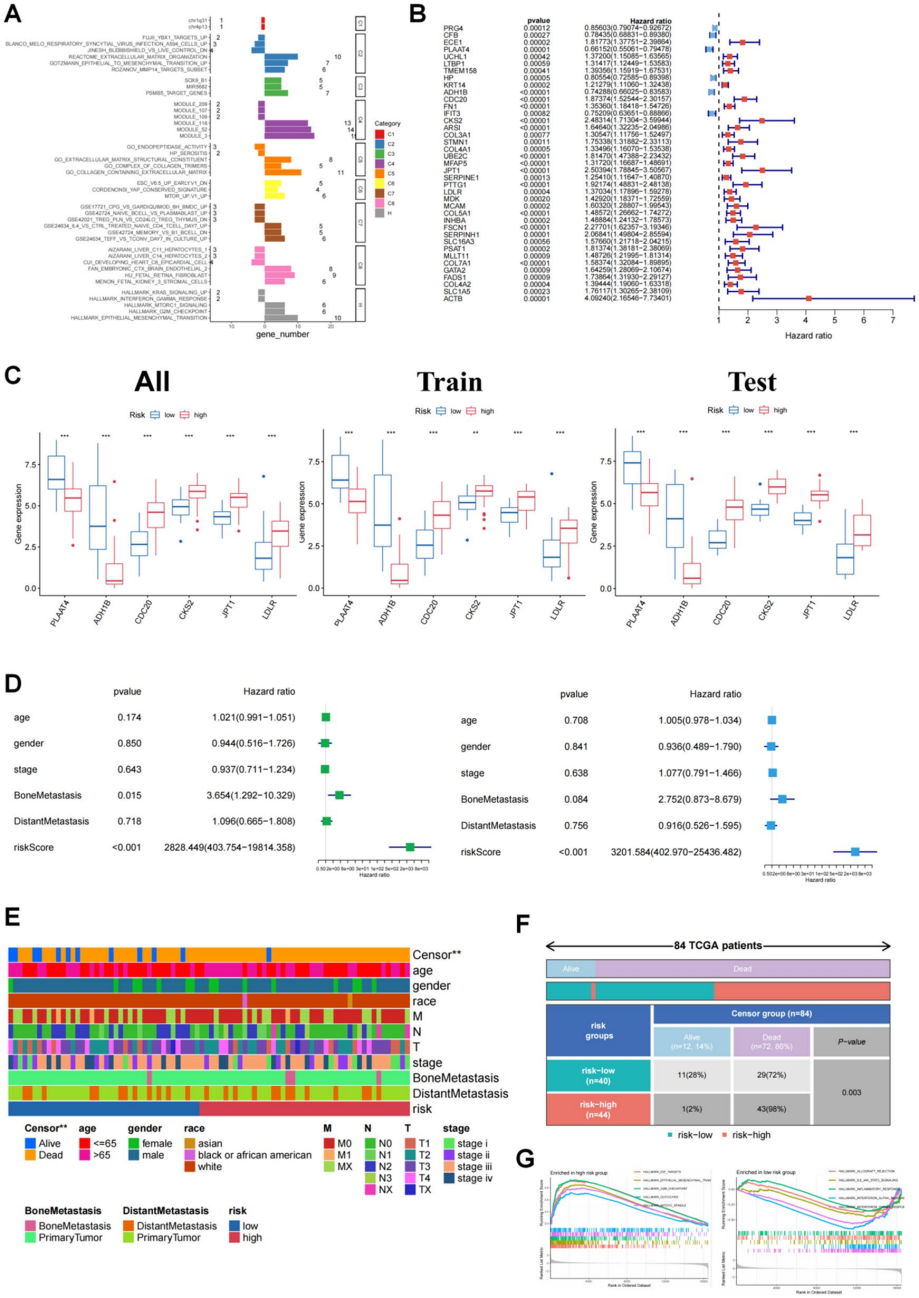
Construction of prognostic prediction model.** A** Over-representation analysis of 39 FDGs in the MSigDB gene sets, comparing the enrichment results of primary MESO with bone metastatic tumors. **B** The univariate Cox regression analysis between gene expression and overall survival (OS). **C** The expression levels of PLAAT4, ADH1B, CDC20, CKS2, JPT1 and LDLR, the genes selected to be included in the risk score calculation, in the low-risk group and high-risk group of the all, train and test cohorts. The all cohort referred to the entire TCGA cohort of MESO patients from which the train and test were randomly separated at the ratio of 6:4. **D** The forest plots showed the univariate and multivariate Cox regression analyses for clinical parameters (age, gender, stage, bone metastasis and distant metastasis) and risk score. **E** The clinical parameters’ differences between the low-risk group and high-risk group. **F** The comparison of survival outcomes (alive or dead) between the low-risk group and high-risk group with chi-square test. **G** The gene set enrichment analysis (GSEA) of the high-risk group and the low-risk group, based on the hallmark gene sets

Nine MSigDB gene categories and the corresponding 42 pathways were recognized from FDGs in ORA analysis of the primary MESO and bone metastatic tumors, as shown in Fig. 5A. The hazard ratios for 39 FDGs were included in Fig. 5B based on a univariate Cox regression analysis between gene expression and related overall survival (OS), identifying six protective factors (ADH1B, CFB, PRG4, PLAAT4, HP, and IFIT3) and 33 hazardous factors with statistical significance (p < 0.001). In order to make the best use of the relationship between FDGs and survival, we applied Lasso regression analysis to select optimal genes from these FDGs for constructing a prognostic prediction model, decreasing bias, and finally including six key FDGs in the risk score formula (Additional file 1: Fig. S9A, B). The whole cohort of MESO was randomly divided into a train cohort and a test cohort at a ratio of 6:4 to validate the model internally. According to the individual risk score, patients in the three cohorts (all, train, and test cohorts) were segmented into a high-risk group and a low-risk group. The distribution of the two groups in each cohort was shown in Additional file 1: Fig. S9C. Among the six key FDGs, ADH1B and PLAAT4 (protective factors) were generally highly expressed in the low-risk group, while the expression of CDC20, CKS2, IPT1, and LDLR (hazardous factors) was relatively high in the high-risk group (Fig. 5C and Additional file 1: Fig. S9D). It was not surprising that the survival condition of low-risk was distinctly superior to the high-risk score in the three cohorts (Additional file 1: Fig. S9E), which was in accord with the phenomenon that the survival time showed a downward trend with the increase in risk score (Additional file 1: Fig. S9F). With ROC curves and the areas under the curves (AUC = 0.916 in the all cohort, AUC = 0.911 in the train cohorts, and AUC = 0.914 in the test cohort), the model also displayed excellent sensitivity and specificity (Additional file 1: Fig. S9G). Further, as depicted in Fig. 5D, we performed univariate and multivariate Cox regression analyses for age, gender, stage, bone metastasis, and risk score and proved that risk score could be regarded as a prognostic factor independent of these clinical measures (p < 0.001 in both Cox regression analyses; HR = 2828.449 in the univariate model and HR = 3201.584 in the multivariate model). Via a comparison of some clinical characteristics between the low-risk group and the high-risk group, we found that more patients were alive in the low-risk group (p = 0.003) despite the high mortality in both groups (Fig. 5E, F). In the GSEA analysis of both groups, pathways related to cell cycle and migration, such as the G2M checkpoint, E2F targets, and EMT, appeared to be active in the high-risk group, which might be the reason for the poor prognosis (Fig. 5G). Moreover, the contrast in immune infiltration between the two groups provided some information on the prognosis difference (Additional file 1: Fig. S10A–C). Survival analyses were carried out for immune cells and components that had statistically significant differences between the two groups (Additional file 1: Fig. S10D). The result demonstrated that the resting mast cells, plasma cells, neutrophils, and Treg T cells, which were highly infiltrative in the low-risk group, were connected to the higher survival probability with a p < 0.05. High aggregation of M1 and M2 macrophages, APC-co-inhibition, and APC-co-stimulation, on the other hand, were associated with a lower survival probability (p < 0.05).

### The potential regulatory mechanism of LDFM, HDFM and MDFM

**Fig. 6:**
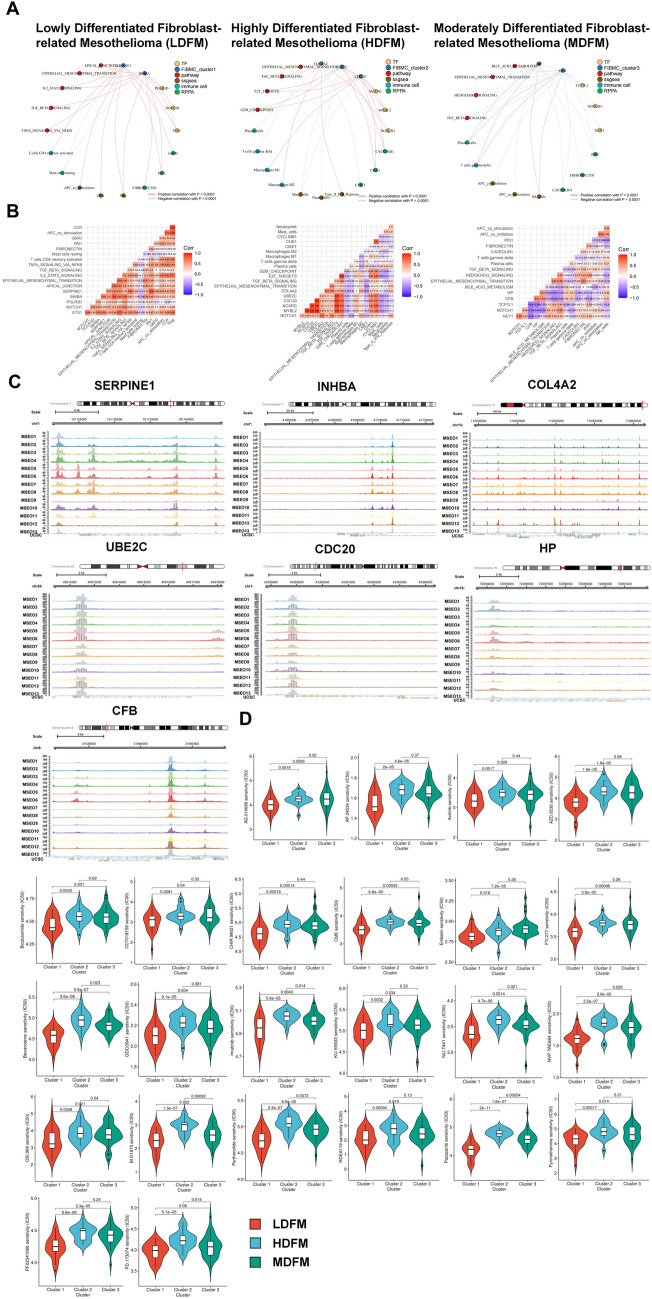
The prediction of regulatory mechanism in each subtype.** A** The regulatory networks of each MESO subtype, containing critical FDGs (blue circles), TFs (yellow circles), pathways (red circles), immune cells (blue-green circles), immune components (brown circles), and RPPA (green circles). The red lines between circles signaled positive correlation and the blue ones symbolized negative correlation with p < 0.0001. **B** The co-expression heatmaps reveal the interrelationship between components in each regulatory network. **C** The ATAC-seq results of the FDGs in the regulatory networks. **D** The violin plots demonstrated the IC50 distribution of the predicted 24 inhibitors among 3 different subtypes

In order to investigate the possible functions and molecular mechanisms of FDGs in the development of MESO, integrated regulatory networks were created, identifying crucial transcription factors (TFs), pathways, immune cells, immune components, and RPPA that were associated with marker FDGs in the each MESO subtypes (Fig. 6A). Heatmaps were used to depict the correlation situations of regulatory network elements with one another (Fig. 6B).

#### MESO subtype 1: lowly differentiated fibroblast-related mesothelioma (LDFM)

SERPINE1 and INHBA, two risk genes related to differentiated state 1 (Fig. 4A), appeared in the regulatory network of LDFM (Fig. 6A), showing positive correlation with several signaling pathways, including apical junction, EMT, IL2_STAT5 signaling, TGF_beta signaling, and TNFA_signaling via NFκB. The expression level upregulation of the genes might be mediated by TFs, such as POLR3D, NOTCH1 and ETS1, which were widely expressed at high levels in bone metastatic tumor samples (Additional file 1: Fig. S11A). The ATAC-seq analysis revealed increased chromosome accessibility near SEPINE1 and INHBA, suggesting potential for binding of TFs or other regulatory elements to these regions (Fig. 6C). Also, the negative association of SERPINE1 and INHBA with resting mast cells that was shown to be favorable for survival in immune infiltration analysis (Additional file 1: Fig. S10D) and the positive association with APC_co_stimulation which were detrimental to survival, suggested the malignant regulatory mechanism of these two genes in LDFM. In addition, with the enrichment of some energy metabolic pathways (Additional file 1: Fig. S11B), the hypermetabolic trait of LDFM was unveiled, laterally reflecting the highly malignant nature of this subtype.

#### MESO subtype 2: highly differentiated fibroblast-related mesothelioma (HDFM)

In the regulatory network of HDFM (Fig. 6A), UBC2E and CDC20 had a positive relationship with the E2F target and G2M checkpoint, in agreement with the pathway enrichment result that HDFM was enriched in the cell cycle pathway (Additional file 1: Fig. S11B), hinting the highly proliferative ability. Another FDG in the network, COL4A2, was tied to EMT and TGF_beta signaling. The linkage between these three FDGs and immune components indicated that they might promote some immune infiltration that was inimical to survival. Moreover, chromosomal accessibility was also increased in regions close to these three genes (Fig. 6C), which was possibly in association with the three TFs (NCAPG, MYBL2 and NOTCH1) in the regulatory network.

#### MESO subtype 3: moderately differentiated fibroblast-related mesothelioma (MDFM)

Dissimilar to the genes identified in the LDFM and HDFM-related regulatory networks, both HP and CFB in the MDFM regulatory network were regarded as favorable factors for survival and associated with differentiation state 3 (Figs. 4A and 5B). Their negative connections with pathways, including EMT, hedgehog and TGF_beta signaling, also affirmed their advantageous role. At the same time, we found that their expression was also significantly lower in bone metastases than in primary tumors (Additional file 1: Fig. S11A), implying that the expression deficiency or downregulation of HP and CFB might lead to the augmented invasion of tumors. Actually, the expression of these two FDGs was higher in HDFM than in MDFM, as illustrated in Fig. 4D and Additional file 1: Fig. S5C, which partly explained the best prognosis of HDFM among the three subtypes.

### Prediction of inhibitors

For increasing drug options to improve the survival of MESO patients with poor prognosis, we estimated the sensitivity of the three subtypes to various inhibitors by leveraging the pRRophetic R package. 24 inhibitors were finally filtered out, to which LDFM was more sensitive than HDFM (Fig. 6D), which were AG.014699, AP.24534, axitinib, AZD.0530, Bicalutamide, CCT018159, CCIR.99021, CMK, Embelin, FTI.277, Bexarotene, GDC0941, Imatinib, KU.55933, NU.7441, NVP.TAE684, OSI.906, BI.D1870, Parthenolide, RDEA119, Pazopanib, Pyrimethamine, PF.02341066, and PD.173074.

### Clinical specimen validation

**Fig. 7:**
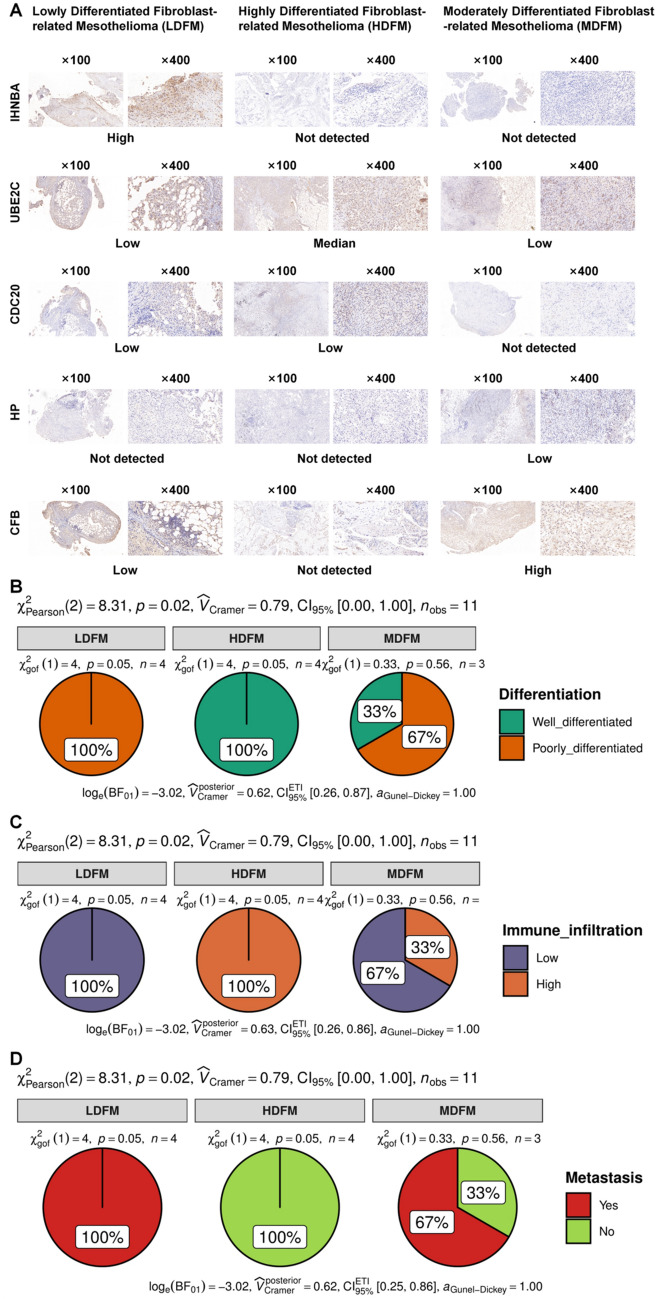
Clinical specimen validation.** A** The immunohistochemical staining slides of the five different markers, INHBA, UBE2C, CDC20, HP and CFB in ×100 and ×400 fields of the light microscope in each MESO subtype. **B** A significant difference was revealed in the differentiation of LDFM, HDFM, and MDFM subtypes (p = 0.02). **C** A significant difference was obtained in the immune infiltration of LDFM, HDFM, and MDFM subtypes (p = 0.02). **D** A significant difference was shown in the metastasis of LDFM, HDFM, and MDFM subtypes (p = 0.02)

To further validate the clinical subtyping of MESO patients into LDFM, HDFM and MDFM, clinical specimens were enrolled for wet experiment validation. The immunohistochemical staining slides of the five different markers, INHBA (LDFM), UBE2C (HDFM), CDC20 (HDFM), HP (MDFM), and CFB (MDFM) in ×100 and ×400 fields of the light microscope were visualized in Fig. 7A. Subsequently, Pearson Chi-square tests were carried out to demonstrate significant differences in the differentiation (p = 0.02, Fig. 7B), immune infiltration (p = 0.02, Fig. 7C) and metastasis (p = 0.02, Fig. 7D) among LDFM, HDFM, and MDFM subtypes. The details were shown in Additional file 1: Table S2. From that, we could deduce that HDFM indicated good differentiation, high immune infiltration, and non-metastasis of MESO, while LDFM suggested poor differentiation, low immune infiltration, and metastasis of MESO, with MDFM in the middle, which was a decent validation for our clinical classification of MESO patients.

## Discussion

Since the potent efficacy of single-cell RNA sequencing to anatomize cellular heterogeneity was demonstrated in several articles [26–28], we applied it to identify fibroblast subtypes in MESO. Among the seven subtypes, the KRT19_FIB which was related to the low differentiation in the pseudotime analysis, predominated in the interaction between fibroblast subtypes. The ANXA1 and CALM2 expressed in KRT19_FIB, were linked to EGFR on itself and the other six subtypes (Fig. 2F). The epidermal growth factor receptor (EGFR), a member of the ERBB family of tyrosine kinase receptors, can be activated by binding to specific ligands and then initiate the downstream signaling to control cell proliferation and differentiation [29]. Ganggang Mu et al. have noted that a high level of Calmodulin 2 (CALM2), which belongs to the family of calmodulin genes, can motivate tumor metastasis via the JAK2/STAT3/HIF-1/VEGFA axis and enhance proliferation, migration, and polarization of macrophages [30]. Meanwhile, CALM1, which together with CALM2 encodes calmodulin (CALM), has been reported to have synergistic effects with EGFR in promoting tumor metastasis, resulting in a poor prognosis [31]. Thus, we speculate that KRT19_FIB may achieve regulation of proliferation and differentiation of other fibroblast subtypes as well as self-regulation through the binding of CALM2 and EGFR.

Next, we screened out 39 genes defined as fibroblast differentiation-related genes (FDGs) that were linked to both fibroblast differentiation and patient survival. Living up to our expectations, the classification constructed on the basis of 39 FDGs effectively distinguished three MESO subtypes with prognostic differences, which was further validated by immunohistochemical staining and statistical analysis by clinical specimens.

Among the three subtypes, LDFM with high expression of genes in differentiation state 1 featured the worst prognosis. As the marker FDG of LDFM, INHBA is one of the TGF-β superfamily members. Mounting evidence shows that the overexpression of INHBA is prevalent in various cancers and that it may serve as an oncogene [32–34]. The mechanism of its protumor function involves the INHBA-induced EMT, mediated by the activating TGF-β signaling pathway [35]. In the SMAD-independent way that can be found in MESO [36, 37], TGF-β activates the downstream effector, SMAD, subsequently upregulates some EMT-related TFs, including SNAIL, SLUG, ZEB1 and TWIST, and ultimately triggers EMT [38]. Except for the INHBA-TGF-β-EMT axis, the apical junction, another pathway positively linked to INHBA, may be concerned in EMT as well, as the absence of adherens junction protein E-cadherin, claudin-1, -2 and occludin has been discovered in TGF-β-induced EMT [39]. Consequently, LDFM may be a highly invasive subtype with a tight junction with EMT. Moreover, targeting INHBA may be able to attenuate the activation of fibroblasts in the stroma [40]. Collectively, INHBA is a promising target for treatment in the future.

The survival condition of HDFM was noticeably better than that of the other two subtypes, partly due to the high expression of favorable genes such as CFB and HP, which were downregulated in bone metastatic tumors (Additional file 1: Fig. S8B and Fig. S11A) and might suppress EMT, hedgehog, and TGF-signaling, as shown in the network of MDFM (Fig. 6A). Nevertheless, its prognosis is still not optimistic, and novel treatment strategies should be investigated. The two marker FDGs in HDFM, CDC20 and UBE2C had been previously reported to have a strong correlation in 27 cancers [41]. Corresponding to their positive correlation with the E2F target and the G2M checkpoint (Fig. 6A), both of them are responsible for sustaining the normal performance of the cell cycle. CDC20, also known as cell division cycle 20 homologue, encoded by the gene CDC20, is the co-activator of the anaphase-promoting complex (APC), and their complex, APC^Cdc20^, targets cyclin B, whose accumulation and degradation actuate the G2-to-M shift and mitotic exit, respectively [42]. There is also evidence that APC^Cdc20^ can induce the destruction of E2F1, which is an E2F transcription factor that manages the G1-to-S transition, in mitotic phase [43, 44]. With high expression in diverse cancers, it has been recognized as a prognostic biomarker, associated with poor outcomes, in epithelial ovarian cancer [45], papillary renal cell carcinoma [46], colorectal cancer [47] and so on. UBE2C, encoding the ubiquitin-conjugating enzyme E2C, is related to the G2/M phase [48]. Moreover, UBE2C may be regulated by E2F1, as binding sites of the TF have been found on the promoter and enhancer regions of gene UBE2C [41]. Similar to CDC20, overexpression of UBE2C has been observed in different cancers, fueling cancer cell proliferation [49–51]. Thus, targeting CDC20 and UBE2C has the potential to impede the malignant cell proliferation of MESO by repressing the cell cycle.

We also generated a prognostic prediction model with robust assessment capability (AUC > 0.910). The model was based on the risk score algorithm, which centered around the expression degree of six key FDGs (ADH1B, PLAAT4, CDC20, CKS2, IPT1, and LDLR). ADH1B and PLAAT4 were identified as favorable genes for survival in our study. As a drug metabolism-related gene, the high level of ADH1B expression has been reported to be connected to a good prognosis in ovarian cancer, along with its positive correlation with multiple immune cells [52]. As for PLAAT4, whose another familiar name is RARRES3, it is commonly believed to function as a tumor suppressor and is related to tumor differentiation [53, 54]. The downregulation of RARRES3 can enhance the attachment of malignant cells to lung parenchyma in lung metastases [55]. In bladder cancer, knockdown of KDM2A which is capable of restraining RARRES3, diminishes high-grade bladder cancer cell growth, aggressiveness, and spheroid formation [56]. Among the remaining four harmful genes, as well as CDC20 mentioned before, CKS2 is an influential gene in the cell cycle, encoding cyclin-dependent kinase subunit 2. The high CKS expression predicts poor survival for many malignancies [57–59], and the co-expression of CKS and some immune checkpoints, including PD-1 and CTLA4, has been discovered in glioma [60]. Additionally, in complex with SSBP1, CSK2 is conjected to take part in mitochondrial DNA (mtDNA) replication, accelerating tumor invasion [61]. Since the formation of steroid hormones and cellular membranes both entail the presence of cholesterol, tumors characterized by uncontrollable proliferation often demand more cholesterol. The low-density lipoprotein receptor (LDLR), encoded by the gene of the same name, governs the absorption of the majority of necessary fatty acids and cholesterol. Numerous studies have demonstrated that the expression of LDLR and the uptake of LDL-C are amplified in a variety of cancer cells, including colorectal cancer [62], breast cancer [63], and lung cancer [64]. Therefore, referring to the previous investigation results, it is also plausible that these FDGs were selected as the cornerstones for building the prognostic model.

Although we achieved ideal results in the present study, limitations still existed, admittedly. Firstly, the majority of the work in the study relied on bioinformation tools, which signified that results were lacking in experimental verification, such as cell-to-cell interactions, immune infiltration, and gene regulatory networks. Then, as we integrated single-cell sequencing with bulk RNA sequencing data which lacked the capacity to discriminate between distinct cellular populations residing within the tumor microenvironment, to explore the significance of FDGs in the progression of MESO tumors in the study, further experimental analysis should be required to elucidate the specific mechanisms of FDGs expression in fibroblasts and their role in MESO tumor action. Moreover, since the validation of the prognostic model was performed with internal cohorts, additional external validation is necessary to ascertain the universality of the developed model. Additionally, to conclusively confirm the effects of inhibitors on MESO subtypes, further cellular and animal experiments and clinical trials should be required and possibly included in our future work. Eventually, due to the rarity of MESO as a malignancy, the clinical specimens collected for validation is limited, and augmenting number of MESO specimens and sequencing data from these specimens for more persuasive validation and further exploration holds substantial promise.

## Conclusion

Integrating sc-RNA sequencing and bulk RNA sequencing data, we acquired 39 fibroblast differentiation-related genes (FDGs), relied on which we constructed an innovative classification (fibroblast differentiation-based classification, FDBC) and prognostic prediction model of MESO independent of clinical parameters, with sound prognostic performance, to facilitate the precise diagnosis and prognostic judgement. To further investigate the significance and functions of FDGs, multi-omics and mechanistic exploration-related bioinformation analysis were also merged into the study, providing some prospective therapeutic targets to be researched in future studies. We believe that our study is meaningful in advancing future clinical and fundamental research on MESO.

### Supplementary Information

**Additional file 1: Figure S1.** The analysis workflow of the study.** Figure S2.** Characters of the seven cell types (the supplement to Fig. 1). **Figure S3.** Supplementary materials for Fig. 3. **Figure S4.** The Kaplan–Meier curves of the 39 FDGs. **Figure S5.** Supplementary materials for Fig. 4. **Figure S6.** Muti-omics analysis based on the bulk RNA-seq profiles in TCGA database in association with the PCA score. **Figure S7.** Muti-omics analysis based on the bulk RNA-seq profiles in TCGA database in association with the PCA score. **Figure S8.** Differential expression analysis of genes, transcription factors (TFs) and pathways between the primary MESO and bone metastatic tumor. **Figure S9.** Supplementary materials for Fig. 5. **Figure S10.** Immune infiltration analysis in the high-risk group and low-risk group. **Figure S11.** Supplementary materials for Fig. 6. **Table S1.** The results of Cox regression analysis and Kaplan–Meier survival analysis of the 39 FDGs. **Table S2.** Clinical and pathological information of validation samples.

## Data Availability

The datasets generated and/or analysed during the current study are available in the the NCBI-GEO, TCGA and MET-500 database.
