# Supplementary material for: A gene signature linked to fibroblast differentiation for prognostic prediction of mesothelioma
Source: Cell Biosci. 2024 Mar 10;14:33. doi: 10.1186/s13578-023-01180-7 (PMC10926647; doi:10.1186/s13578-023-01180-7)
Supplement: Supplementary file 1 — Additional file 1: Figure S1. The analysis workflow of the study. Figure S2. Characters of the seven cell types (the supplement to Fig. 1). Figure S3. Supplementary materials for Fig. 3. Figure S4. The Kaplan–Meier curves of the 39 FDGs. Figure S5. Supplementary materials for Fig. 4. Figure S6. Muti-omics analysis based on the bulk RNA-seq profiles in TCGA database in association with the PCA score. Figure S7. Muti-omics analysis based on the bulk RNA-seq profiles in TCGA database in association with the PCA score. Figure S8. Differential expression analysis of genes, transcription factors (TFs) and pathways between the primary MESO and bone metastatic tumor. Figure S9. Supplementary materials for Fig. 5. Figure S10. Immune infiltration analysis in the high-risk group and low-risk group. Figure S11. Supplementary materials for Fig. 6. Table S1. The results of Cox regression analysis and Kaplan–Meier survival analysis of the 39 FDGs. Table S2. Clinical and pathological information of validation samples. [file 13578_2023_1180_MOESM1_ESM.docx]

**Additional Figures**

**
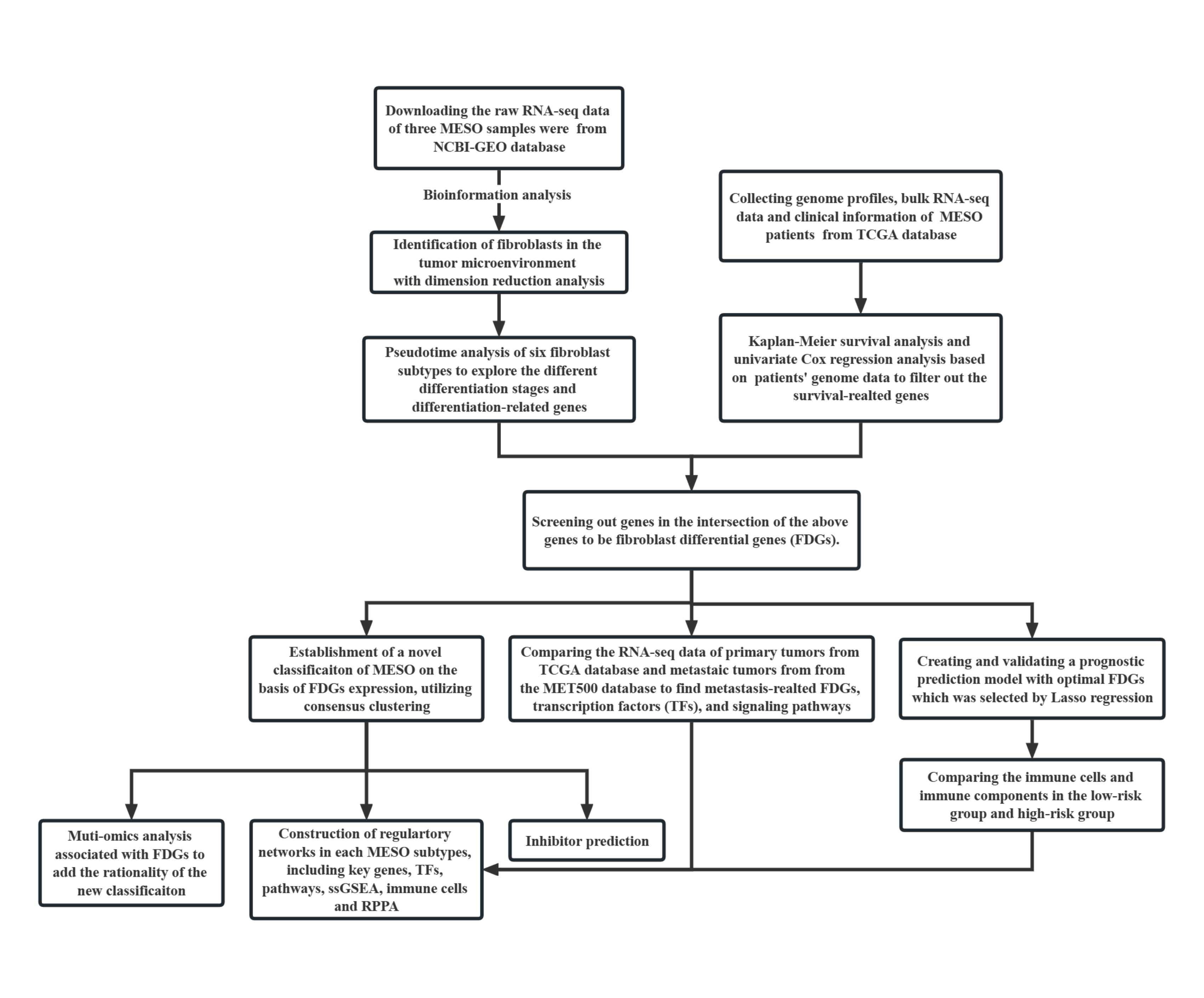
**

**Figure S1.** The analysis workflow of the study.

**
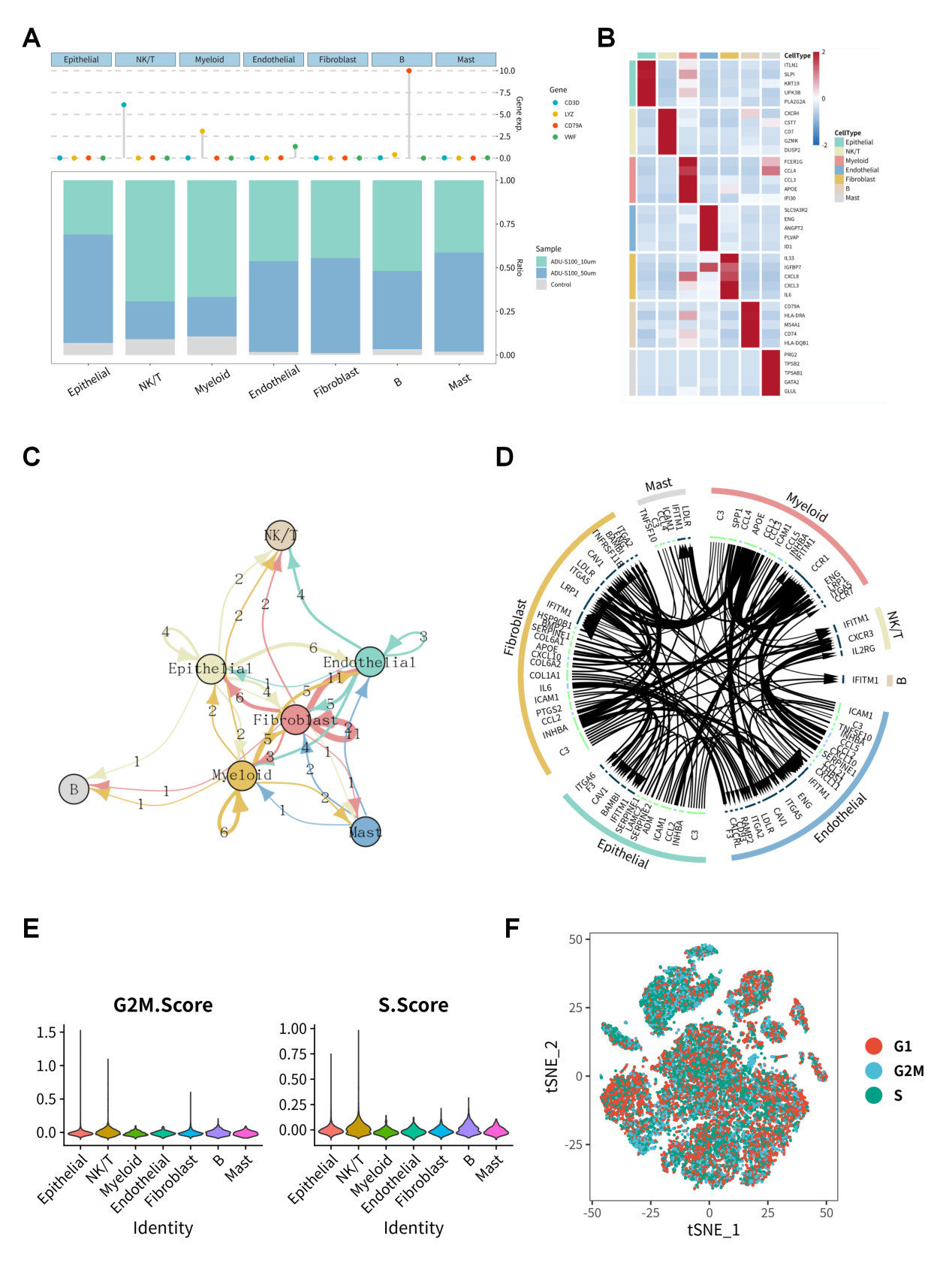
Figure S2. Characters of the seven cell types (The supplement to Figure 1).**

**(A)** The Cleveland's dot plot of the expression of four classic cell markers (CD3D, LYZ, CD79A, and VWF) in each cell type, combined with the histogram which shows the proportions of the three samples in each cell type.

(B) The top five differential expressed genes in each cell type.

**(C)** The cell communication network, visualizing the connections between each cell type. The circles with different colors referred to different cell subtypes and the width of lines between cells represents the intensity of communication.

**(D)** The ligand-receptor interactions, predicting the possible interactions between each cell type. The ligands lie at the beginnings of the lines and the arrows point to the receptors.

**(E-F)** The cell cycle scores (G2M. score and S.S score) and cell cycle distribution of cells in the tSNE plot.

**
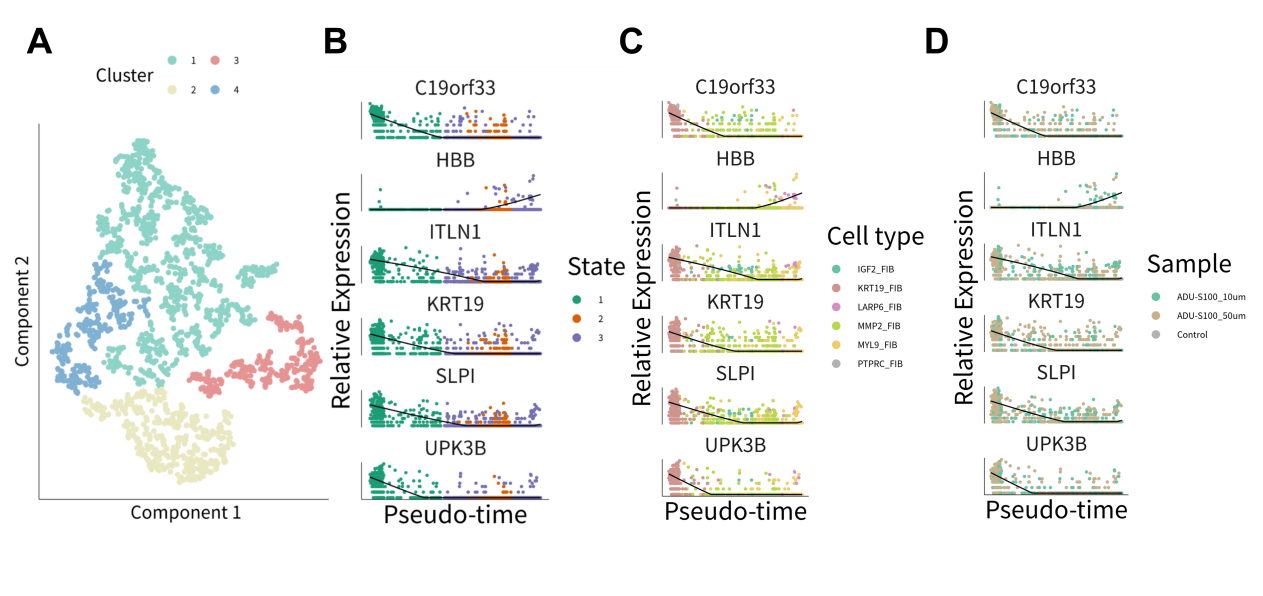
Figure S3. Supplementary materials for Figure 3.**

(A) The tSNE plot of fibroblasts, labeling cells according to the maturation in Monocle-derived pseudotime.

(B) The dynamic expression level of the six representative differentially expressed genes over pseudotime. The differentiation states are marked with different colors.

(C) The dynamic expression level of the six representative differentially expressed genes in the six fibroblast subtypes over pseudotime.

(D) The dynamic expression level of the six representative differentially expressed genes in the three MESO samples over pseudotime.


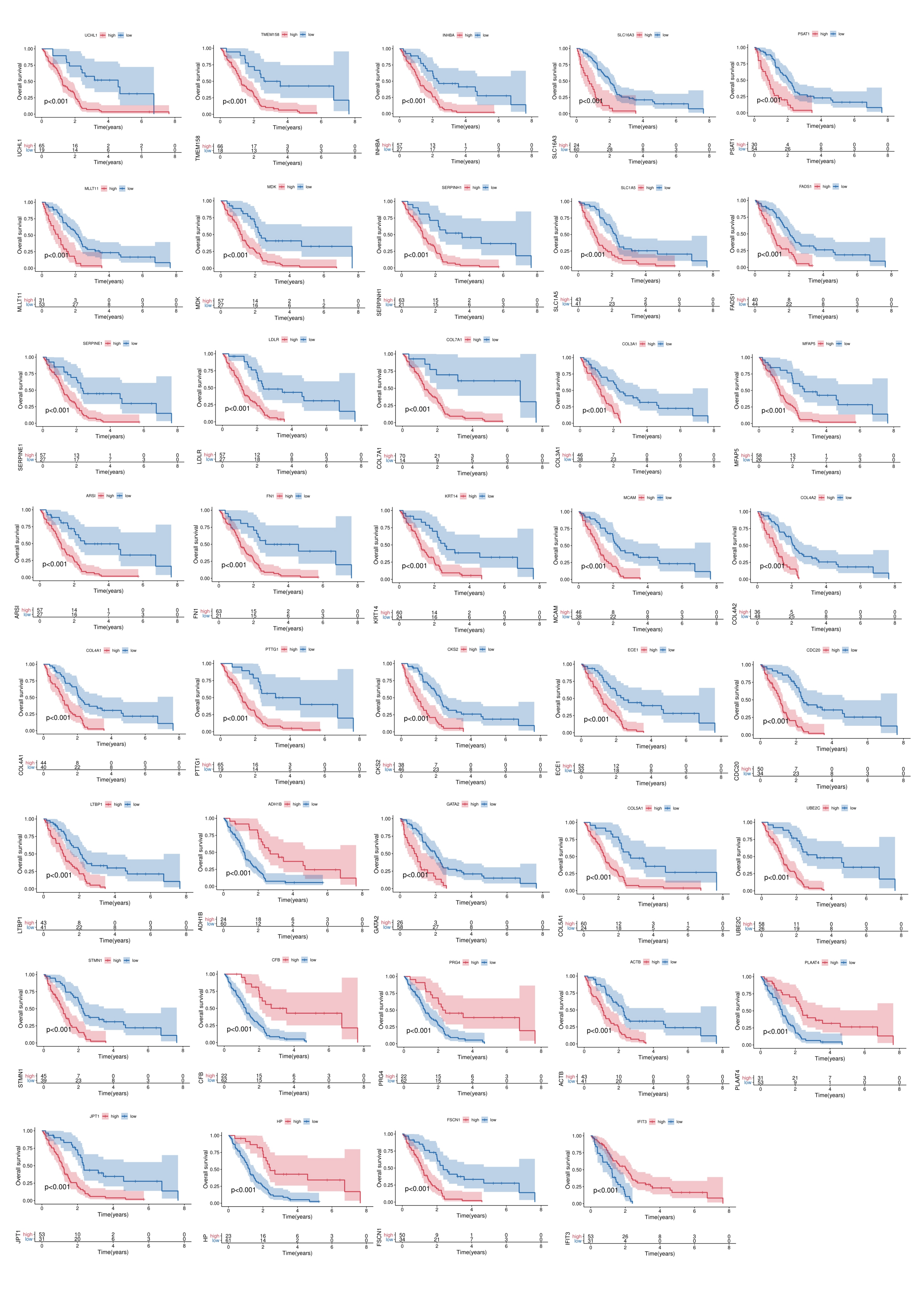


**Figure S4.** The Kaplan-Meier curves of the 39 FDGs.


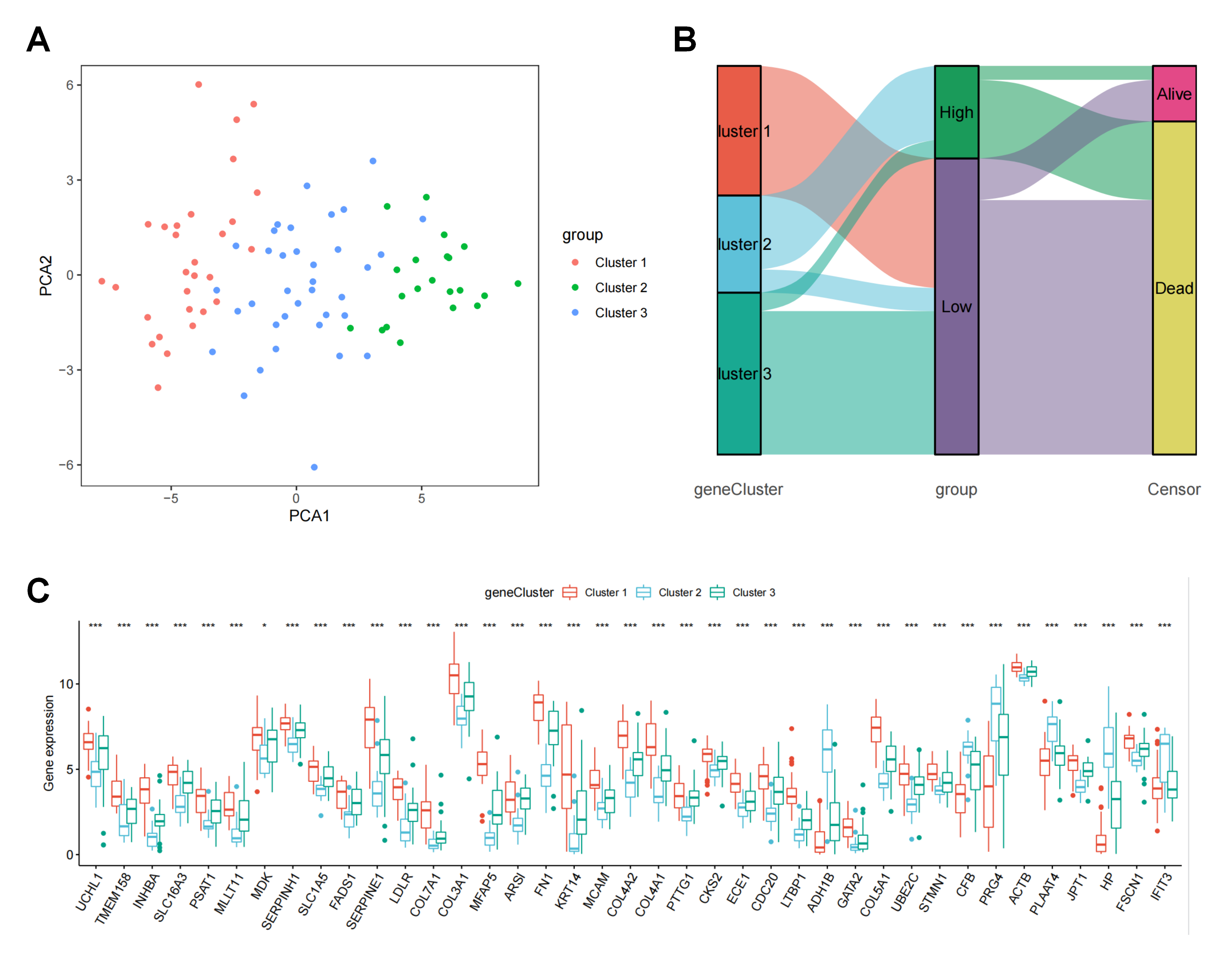


**Figure S5. Supplementary materials for Figure 4.**

(A) The scatter plot of the PCA score of each patient in the three clusters. Colors are utilized to distinguish patients from different clusters.

(B) The Sankey diagram depicts the relationship between clusters, PCA score and survival outcomes.

(C) The expression level comparison of the 39FDGs in the three clusters. “*”: p <0.05, “**”: p< 0.01; “***”: p < 0.001


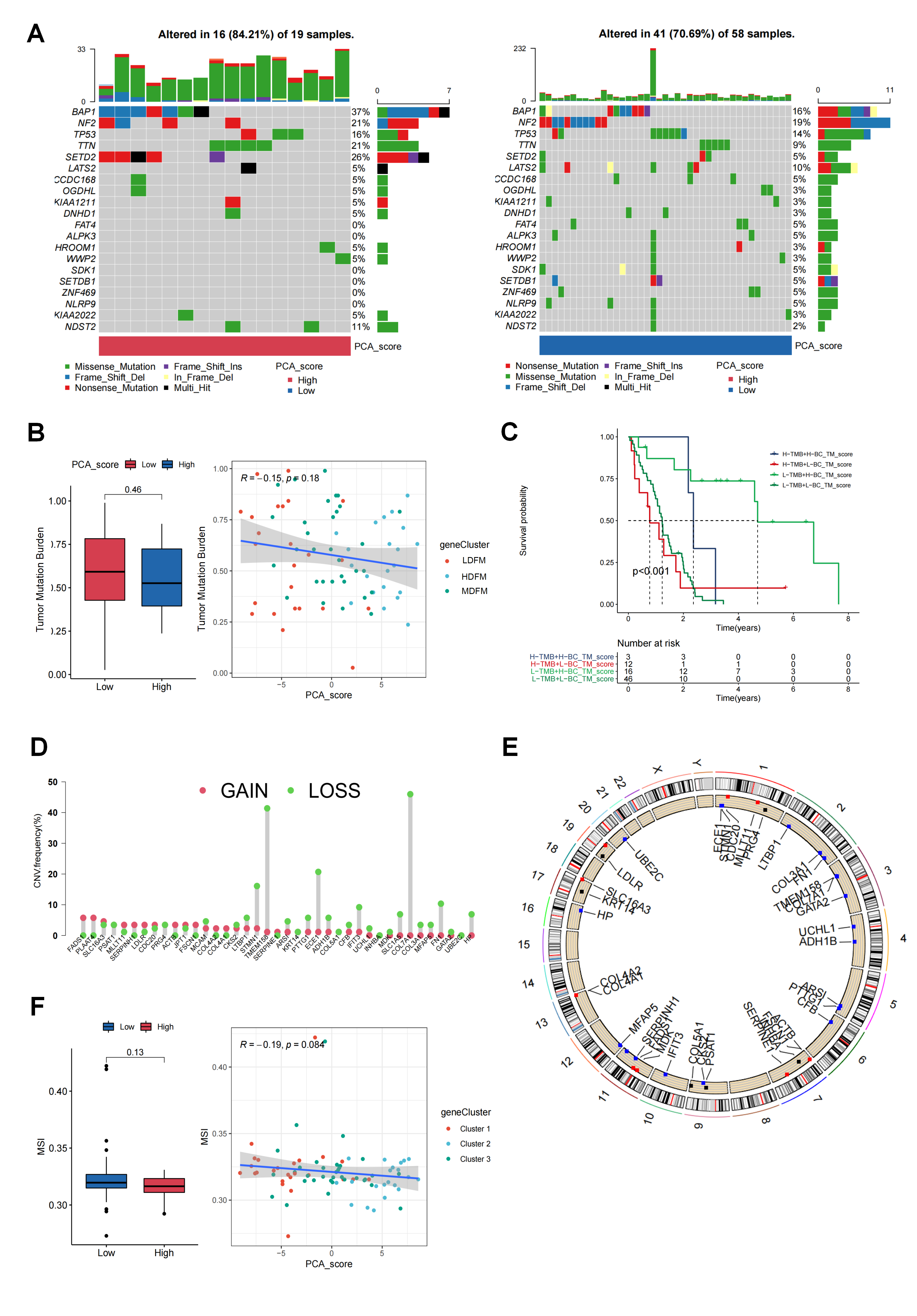


**Figure S6. Muti-omics analysis based on the bulk RNA-seq profiles in TCGA database in association with the PCA score.**

(A) The waterfall plots illustrate the gene mutation landscapes in the high PCA score and low PCA score, providing the prime mutant genes’ types and frequency.

(B) The association between tumor mutation burden (TMB) and PCA score. (r = -0.15, p = 0.18)

(C) The Kaplan-Meier analysis based on the combination of tumor mutation burden and PCA score. H-TMB: high tumor mutation burden; L-TMB: low tumor mutation burden; H-BC_TM_score: high PCA score; L-BC_TM_score: low PCA score.

(D) The Cleverland dot plot of copy number variation (CNV) frequency of FDGs, with CNV increase in red and CNV deletion in green.

(E) The circos plot delineates the location of the FDGs with CNV alternations in the chromosomes.

(F)The association between microsatellite instability and PCA score. (r = -0.19, p = 0.084)


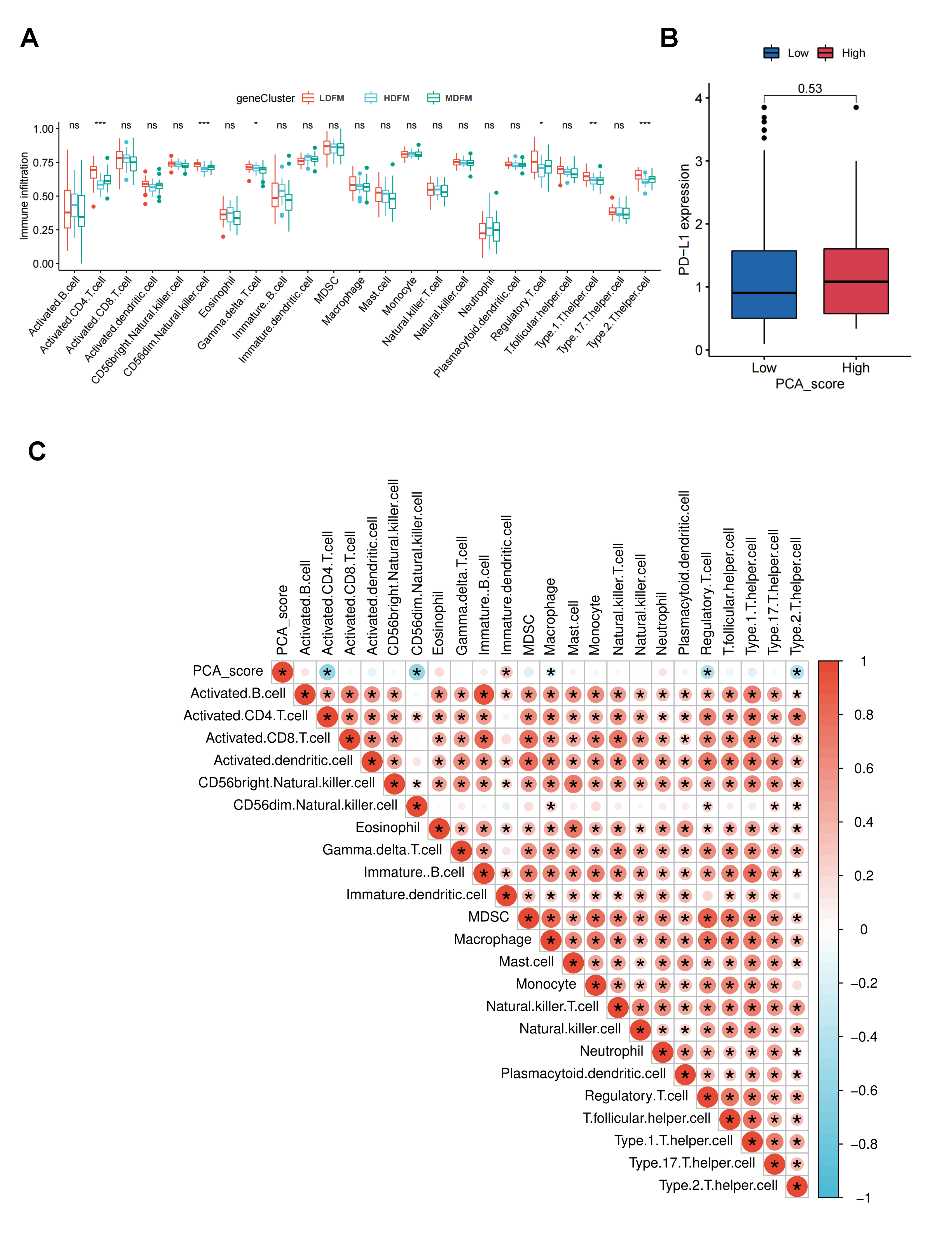


**Figure S7. Muti-omics analysis based on the bulk RNA-seq profiles in TCGA database in association with the PCA score.**

(A) The comparison of 23 immune cell infiltration in LDFM，HDFM and MDFM. ns: no statistical significance; “*”: p <0.05, “**”: p< 0.01; “***”: p < 0.001

(B) The comparison of PD-L1 expression level between the low-PCA score group and the high PCA score group.

(C) The co-expression matrix of the PCA score and the 23 immune cells.


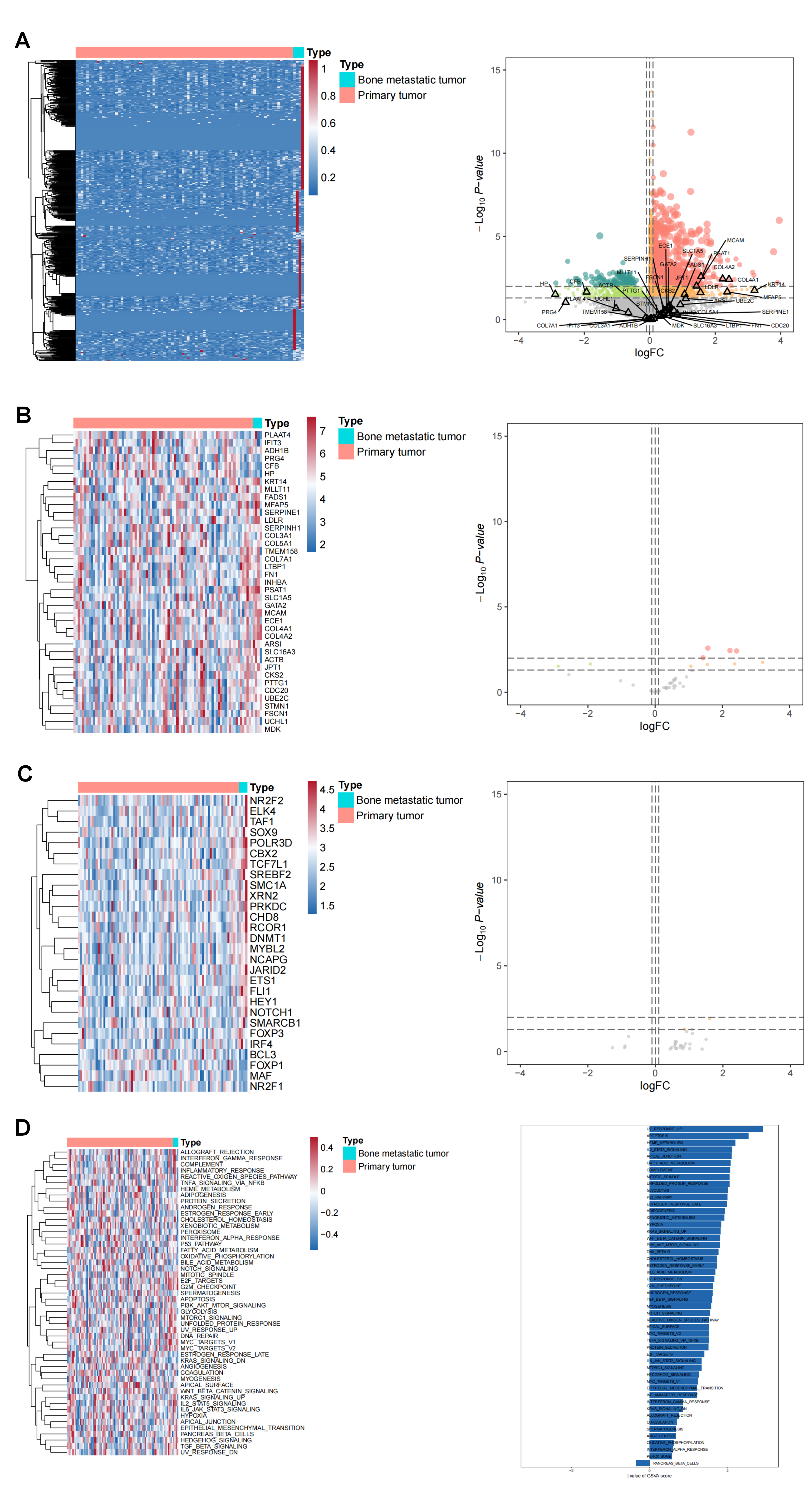


**Figure S8. Differential expression analysis of genes, transcription factors (TFs) and pathways between the primary MESO and bone metastatic tumor.**

(A) The heatmap and volcanic plots of differential expressed genes between the primary MESO and bone metastatic tumors. The red and orange dots in the volcanic plots reveal the up-regulated gens while green and cyan dots indicate the down-regulated genes.

(B) The differential expression analysis of FDGs between the primary MESO and bone metastatic tumors, offering 10 differentially expressed FDGs with statistical significance.

(C) The heatmap and volcanic plots of differential expressed TFs between the primary MESO and bone metastatic tumors.

(D) The contrast of hallmark pathway activities between the primary MESO and bone metastatic tumors with GSVA analysis.


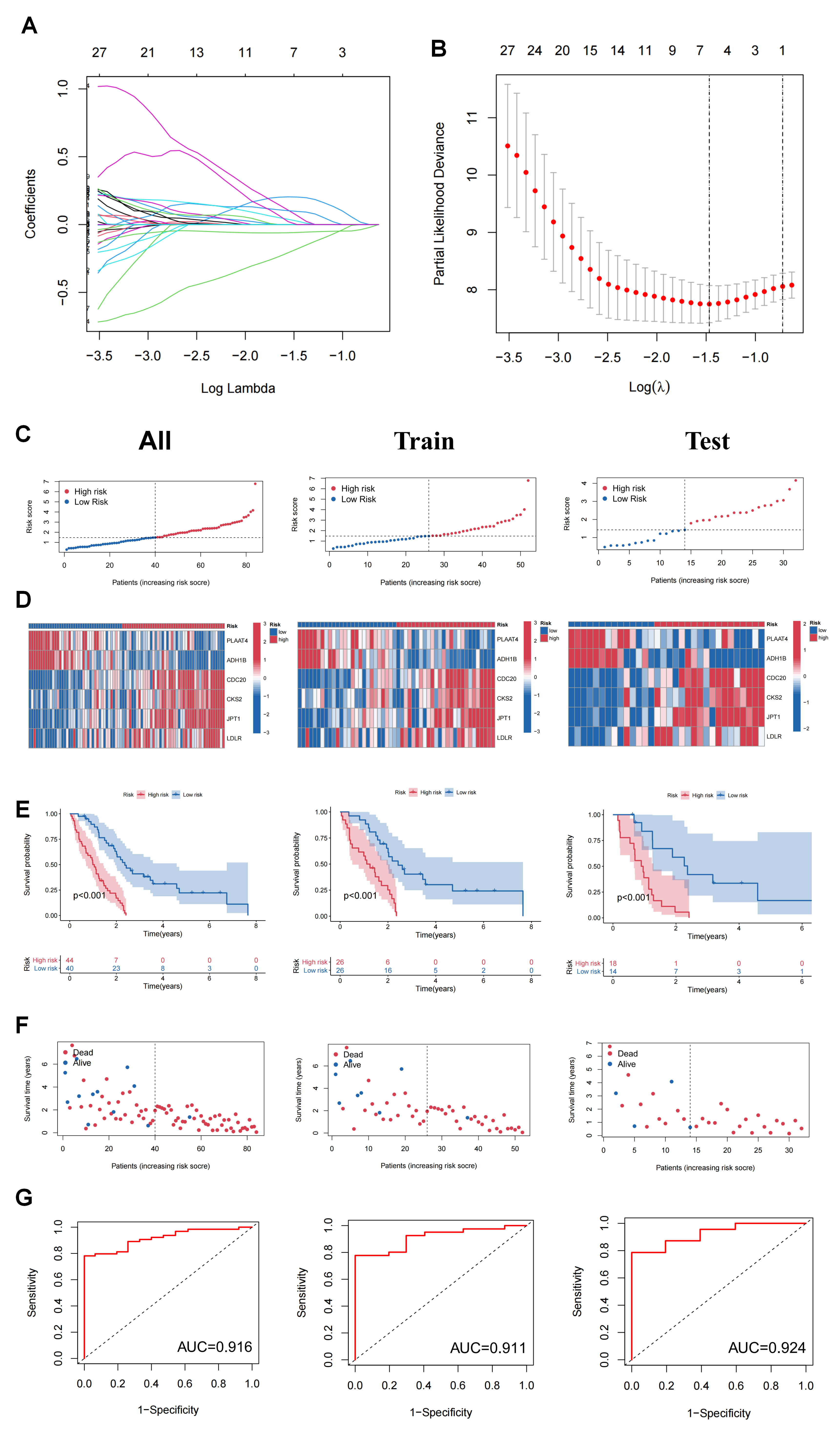


**Figure S9. Supplementary materials for Figure 5.**

(A) The Lasso coefficient profile of the FDGs.

(B) Plot of the ten-fold cross-validation decides the number of key FDGs to create the model and the position of the dotted line figures out the optimal number with the minimum cross-validation error.

(C) The distribution of risk score in the all, train and test cohorts.

(D) The expression heatmaps of the six FDGs which are screened out to construct the prognostic prediction model, illustrate the gene expression discrepancies between the low-risk group and high-risk group.

(E) The Kaplan–Meier survival analysis between the low-risk group and high-risk group in the all, train and test cohorts.

(F) The survival time distribution in the all, train and test cohorts. The red spots represent the dead patient as the blue spots refers to the alive patients.

(G) The ROC curves for FDGs to assess the prognostic prediction model.


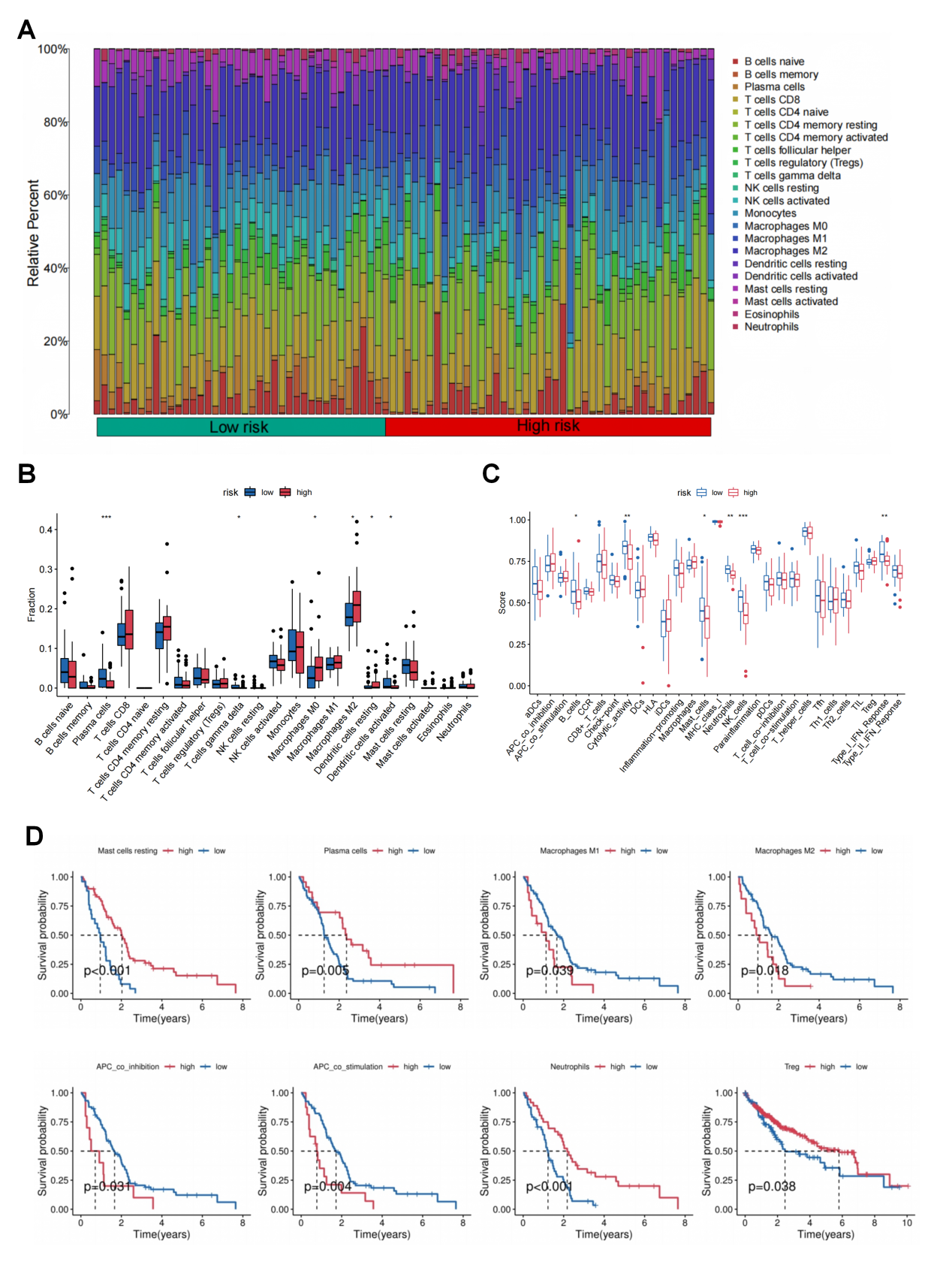


**Figure S10. Immune infiltration analysis in the high-risk group and low-risk group.**

(A) The stacked histogram portrays the composition and proportion of 22 types of immune cells in the low-risk group and high-risk group with CIBERSORT.

(B) Comparison of the immune cell fractions in the high and low groups. “*”: p <0.05, “**”: p< 0.01; “***”: p < 0.001.

(C) Comparison of the immune components in the high and low groups.

(D) The Kaplan–Meier survival analysis of some important immune cells and components.


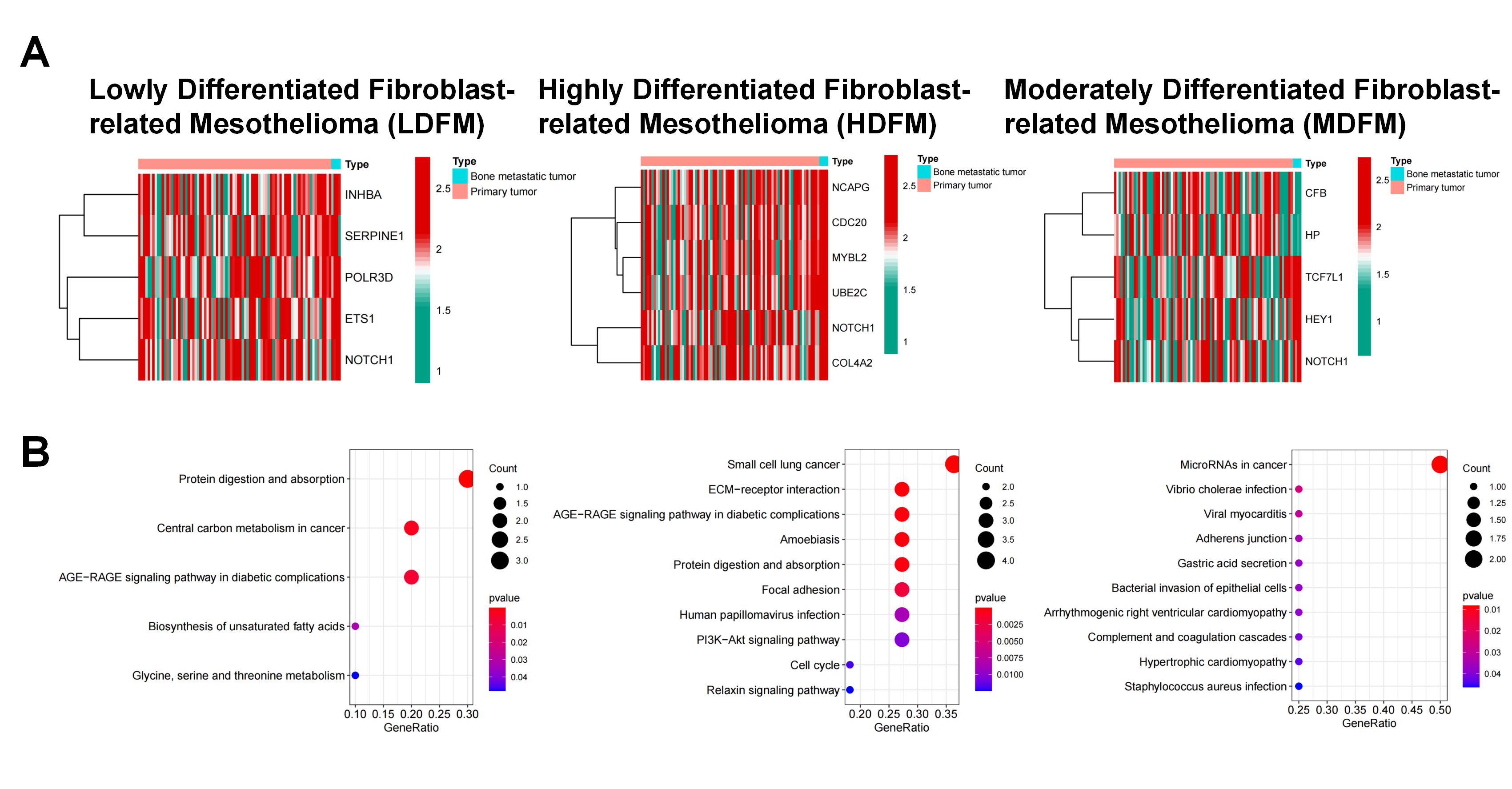


**Figure S11. Supplementary materials for Figure 6.**

(A) The heatmaps compare the expression level of FDGs and TFs in Figure 6A between the primary MESO and bone metastatic tumor.

(B) Bubble maps of the pathway enrichment analysis of each subtype. The circle size denotes the number of enriched genes.

**Table S1. The results of Cox regression analysis and Kaplan-Meier survival analysis of the 39 FDGs.**

| **Gene_id** | **HR** | **HR.95L** | **HR.95H** | **Cox_pvalue** | **KM_pvalue** |
| --- | --- | --- | --- | --- | --- |
| **UCHL1** | 1.372003264 | 1.150851633 | 1.635652158 | 0.000420822 | 2.29E-05 |
| **TMEM158** | 1.393556522 | 1.159191757 | 1.675305029 | 0.000411895 | 5.17E-05 |
| **INHBA** | 1.488844172 | 1.24131975 | 1.785726014 | 1.79E-05 | 3.39E-05 |
| **SLC16A3** | 1.576603818 | 1.217184693 | 2.04215483 | 0.000563066 | 1.12E-07 |
| **PSAT1** | 1.813740863 | 1.381808905 | 2.38068803 | 1.78E-05 | 4.11E-07 |
| **MLLT11** | 1.487256678 | 1.219948103 | 1.813136495 | 8.61E-05 | 1.07E-06 |
| **MDK** | 1.429195452 | 1.183713314 | 1.725586437 | 0.000203996 | 2.16E-05 |
| **SERPINH1** | 2.068405859 | 1.498039211 | 2.855935123 | 1.01E-05 | 1.40E-05 |
| **SLC1A5** | 1.761170211 | 1.302646204 | 2.381092043 | 0.000234815 | 6.20E-05 |
| **FADS1** | 1.738638505 | 1.319295474 | 2.291271297 | 8.58E-05 | 4.20E-05 |
| **SERPINE1** | 1.254099613 | 1.116468492 | 1.408697021 | 0.000134826 | 2.72E-05 |
| **LDLR** | 1.370336672 | 1.178957458 | 1.592782319 | 4.04E-05 | 9.76E-07 |
| **COL7A1** | 1.583736083 | 1.320842941 | 1.898953997 | 6.88E-07 | 4.70E-05 |
| **COL3A1** | 1.305471331 | 1.117564587 | 1.524972618 | 0.000774524 | 8.85E-07 |
| **MFAP5** | 1.317203335 | 1.166867214 | 1.486908368 | 8.36E-06 | 4.96E-06 |
| **ARSI** | 1.646396417 | 1.322347181 | 2.049855892 | 8.25E-06 | 3.39E-06 |
| **FN1** | 1.353598765 | 1.184176324 | 1.547260808 | 9.09E-06 | 1.74E-05 |
| **KRT14** | 1.212785563 | 1.110595128 | 1.324378962 | 1.74E-05 | 5.38E-05 |
| **MCAM** | 1.603201317 | 1.288072973 | 1.995426125 | 2.37E-05 | 6.47E-07 |
| **COL4A2** | 1.394439313 | 1.190595095 | 1.633184117 | 3.73E-05 | 2.91E-07 |
| **COL4A1** | 1.334956835 | 1.160695448 | 1.53538101 | 5.17E-05 | 2.97E-06 |
| **PTTG1** | 1.921736998 | 1.488313702 | 2.48138083 | 5.46E-07 | 1.05E-05 |
| **CKS2** | 2.483136733 | 1.713036128 | 3.599438411 | 1.57E-06 | 8.41E-07 |
| **ECE1** | 1.817731588 | 1.3775084 | 2.398641 | 2.40E-05 | 2.04E-06 |
| **CDC20** | 1.873738149 | 1.525436463 | 2.30156728 | 2.17E-09 | 8.99E-09 |
| **LTBP1** | 1.31416627 | 1.124494723 | 1.535830225 | 0.000591739 | 3.58E-05 |
| **ADH1B** | 0.742875399 | 0.660254896 | 0.835834556 | 7.77E-07 | 1.85E-05 |
| **GATA2** | 1.642585308 | 1.280693899 | 2.106737994 | 9.29E-05 | 1.02E-05 |
| **COL5A1** | 1.485721057 | 1.266623321 | 1.742717841 | 1.15E-06 | 9.71E-06 |
| **UBE2C** | 1.814697271 | 1.473884709 | 2.234317357 | 1.97E-08 | 1.66E-08 |
| **STMN1** | 1.753380225 | 1.318821269 | 2.331128778 | 0.000111362 | 9.04E-07 |
| **CFB** | 0.784354691 | 0.688309391 | 0.893801957 | 0.000267846 | 2.53E-06 |
| **PRG4** | 0.856034704 | 0.790740179 | 0.926720854 | 0.000123072 | 6.94E-05 |
| **ACTB** | 4.092395495 | 2.16546165 | 7.734009459 | 1.43E-05 | 4.83E-06 |
| **PLAAT4** | 0.661523997 | 0.550613373 | 0.794775463 | 1.02E-05 | 1.23E-05 |
| **JPT1** | 2.503938123 | 1.788448508 | 3.505667676 | 9.00E-08 | 5.92E-06 |
| **HP** | 0.805537876 | 0.725845659 | 0.893979679 | 4.73E-05 | 2.13E-05 |
| **FSCN1** | 2.277013977 | 1.623568013 | 3.193455779 | 1.86E-06 | 5.61E-07 |
| **IFIT3** | 0.752091287 | 0.636513101 | 0.888656185 | 0.000818097 | 2.34E-05 |

**Table S2. Clinical and pathological information of validation samples.**

| **Subtype** | **LDFM** | **MDFM** | **HDFM** |
| --- | --- | --- | --- |
| **Number of patients** | 4 | 3 | 4 |
| **Gender** |  |  |  |
| **Male, n（%）** | 2 (50%) | 3 (100%) | 2 (50%) |
| **Female, n（%）** | 2 (50%) | 0 (0%) | 2 (50%) |
| **Age（years）** | 63.25 ± 6.18 | 64 ± 2.65 | 54 ± 19.3 |
| **Differentiation** |  |  |  |
| **Poorly differentiated, n (%)** | 4 (100%) | 2 (66.67%) | 0 (0%) |
| **Well differentiated, n (%)** | 0 (0%) | 1 (33.33%) | 4(100%) |
| **Immune_infiltration** |  |  |  |
| **Low** | 4 (100%) | 2 (66.67%) | 0 (0%) |
| **High** | 0 (0%) | 1 (33.33%) | 4 (100%) |
| **Metastasis, n (%)** | 4 (100%) | 2 (66.67%) | 0 (0%) |
